# Multi-dimensional regulation of LIN-28 temporal expression dynamics in the *C. elegans* heterochronic gene cascade

**DOI:** 10.1242/dev.205391

**Published:** 2026-05-28

**Authors:** Charles Nelson, Victor Ambros

**Affiliations:** Program in Molecular Medicine, University of Massachusetts Chan Medical School, Worcester, MA 01605, USA

**Keywords:** *Caenorhabditis elegans*, Developmental timing, *lin-28*, *lep-5*, microRNA, 3′ UTR

## Abstract

LIN-28 is an evolutionarily conserved RNA-binding protein that is crucial for regulating pluripotency and cell fate determination during animal development. In *Caenorhabditis elegans*, *lin-28* is an integral component of the heterochronic (developmental timing) gene regulatory cascade. Loss-of-function mutations in *lin-28* cause precocious cell fate determination during larval development. Previous studies indicate that proper progression of larval stage-specific cell fates relies on the downregulation of LIN-28, which is negatively regulated by the *lin-4* microRNA through complementary sequences in the *lin-28* 3′ untranslated region (UTR). This study employs CRISPR/Cas9 editing of the endogenous *lin-28* locus to demonstrate that developmental downregulation of LIN-28 involves multiple inputs, including the action of the let-7 family and *lin-4* microRNAs via adjacent complementary sites in the *lin-28* 3′ UTR, along with post-translational inhibition of LIN-28 by the *lep-5* long non-coding RNA, collectively accounting for nearly all LIN-28 repression. Additionally, systematic testing of truncations of the *lin-28* 3′ UTR identifies three positive regulatory regions that enhance LIN-28 expression, counteracting the negative effects of the let-7 and *lin-4* microRNAs and the *lep-5* long non-coding RNA.

## Teaser

**Summary:** This paper reveals multiple regulatory mechanisms that converge on LIN-28 mRNA and protein to shape its developmental expression dynamics in *C. elegans*.

## INTRODUCTION

During animal development, precise gene expression is essential for establishing correct cell lineages and fates. In *Caenorhabditis elegans*, the heterochronic gene regulatory cascade governs the timing of cell fate transitions during larval development through a hierarchical arrangement of genes that restrict or promote these transitions (Rougvie and Moss, 2013). Loss-of-function mutations in the central heterochronic regulators *lin-14*, *lin-28* or *hbl-1* lead to skipping early larval cell fate transitions and precocious expression of later cell fate programs, while gain-of-function alleles cause retarded development due to reiteration of early larval cell fates (Ambros and Horvitz, 1984; Fay et al., 1999; Ilbay and Ambros, 2019).

MicroRNAs are non-coding RNAs that negatively regulate protein expression by base-pairing their ‘seed’ sequence (nucleotides 2-8) with complementary sequences in the 3′ untranslated regions (UTRs) of target messenger RNAs (mRNAs). MicroRNAs with identical seed sequences but different non-seed nucleotides can target the same mRNAs and are classified in the same family (Bartel, 2009; Brancati and Grosshans, 2018; Ambros and Ruvkun, 2018).

In *C. elegans*, the microRNAs miR-48, miR-84, miR-241 and *let-7* microRNA (collectively referred to as let-7 family microRNAs) and *lin-4* microRNA facilitate transitions from early to later larval developmental events. The 3′ UTR regions of the genes *lin-14*, *lin-28* and *hbl-1* contain sequences with full seed complementarity to the *lin-4* microRNA and/or the let-7 family microRNAs (Lee et al., 1993; Moss et al., 1997; Reinhart et al., 2000; Abrahante et al., 2003; Lin et al., 2003). We refer to these microRNA complementary sequences (CSs) as *lin-4*-*CSs* and *let-7-CSs*, noting that ‘*let-7-CS*’ or ‘*let-7* microRNA’ include potential regulation by any or all the let-7 family microRNAs. Similarly, ‘*lin-4-CS*’ or ‘*lin-4* microRNA’ encompasses potential regulation by *lin-4* microRNA itself or its family member, miR-237.

Deletions in the 3′ UTRs of *lin-14* or *hbl-1* that eliminate *lin-4* and *let-7* CSs cause gain-of-function phenotypes (retarded development) that phenocopy the loss of *lin-4* or *let-7* microRNAs, respectively, suggesting that microRNA-mediated repression via the 3′ UTRs of *lin-14* and *hbl-1* is the predominant regulatory mechanism for downregulating LIN-14 and HBL-1 (Wightman et al., 1993; Ilbay and Ambros, 2019).

LIN-28 (Lin28 in mammals; hereafter referred to as LIN-28 unless otherwise noted) is a conserved RNA-binding protein that regulates various mRNAs and noncoding RNAs (Wilbert et al., 2012; Stefani et al., 2015; Kiontke et al., 2019). LIN-28 can bind to the primary and precursor transcripts of the *let-7* microRNA, inhibiting its function by blocking transcript processing and promoting trans-splicing (Rybak et al., 2008; Viswanathan et al., 2008; Stefani et al., 2015; Nelson and Ambros, 2019). Loss of LIN-28 results in misexpression of *let-7* microRNA and premature cellular differentiation (Johnson et al., 2003; Vadla et al., 2012). In wild-type *C. elegans*, LIN-28 expression is highest during the L1-L2 stages and significantly diminishes by the L3 stage (Seggerson et al., 2002). Conversely, expression of *lin-4* microRNA increases during the L1 stage, while levels of *let-7* microRNAs rise during the L2-L4 stages (Feinbaum and Ambros, 1999; Reinhart et al., 2000; Abbott et al., 2005; Nelson and Ambros, 2021).

The *C. elegans lin-28* 3′ UTR contains a *let-7*-*CS* and a *lin-4-CS*, suggesting that the *lin-4* and *let-7* microRNAs inhibit LIN-28 accumulation during later larval stages (Moss et al., 1997; Reinhart et al., 2000). No mutations have been identified in the endogenous *C. elegans lin-28* 3′ UTR, leaving the contributions of the *lin-4* and *let-7* microRNAs to LIN-28 downregulation undetermined. The expression of a high-copy *lin-28* transgene with the *lin-4-CS* deleted results in misexpression of transgenic LIN-28 and gain-of-function phenotypes (Moss et al., 1997). However, the high-copy nature of such transgenes introduces uncertainty about whether these results accurately represent endogenous regulation or levels of LIN-28 expression.

The *lep-5* long non-coding RNA (lncRNA) destabilizes LIN-28 by binding LIN-28 and recruiting the E3 ubiquitin-protein ligase LEP-2 (Herrera et al., 2016; Kiontke et al., 2019). *lep-5* lncRNA expression increases in the L2 stage and it is hypothesized that it eliminates residual LIN-28 that was translated before the expression of the *lin-4* and *let-7* microRNAs (Kiontke et al., 2019). The loss of *lep-5* does not fully derepress LIN-28, indicating that LIN-28 downregulation involves both microRNA-mediated repression and *lep-5* lncRNA-induced destabilization (Kiontke et al., 2019).

Here, we show that the *let-7*-*CS* and *lin-4*-*CS* targeting of LIN-28 mRNA are conserved among *Caenorhabditis* species. We demonstrate that these CSs located in the 3′ UTR of *C. elegans lin-28* function semi-redundantly to downregulate LIN-28 during larval development. Our data suggest that this microRNA-mediated downregulation of LIN-28 works synergistically with the post-translational degradation mediated by the *lep-5* lncRNA. Remarkably, we observed that deleting the entire *lin-28* 3′ UTR results in significantly milder phenotypes compared to deleting only the CSs. This observation led to our identifying three positive regulatory regions within the *lin-28* 3′ UTR that enhance LIN-28 expression by stabilizing its mRNA.

Our findings illustrate that multiple regulatory layers govern LIN-28 dynamics, including mRNA stability, protein synthesis regulation by microRNAs, positive elements in the 3′ UTR and protein stability via the *lep-5* lncRNA. We observe that the heterochronic phenotypes of 3′ UTR mutants can be exacerbated by physiological stresses, suggesting that these regulatory mechanisms are essential for precise LIN-28 expression dynamics, regardless of physiological conditions.

## RESULTS

### The targeting of LIN-28 mRNA by the *let-7* and *lin-4* microRNAs is conserved among *Caenorhabditis* species

**Fig. 1.**
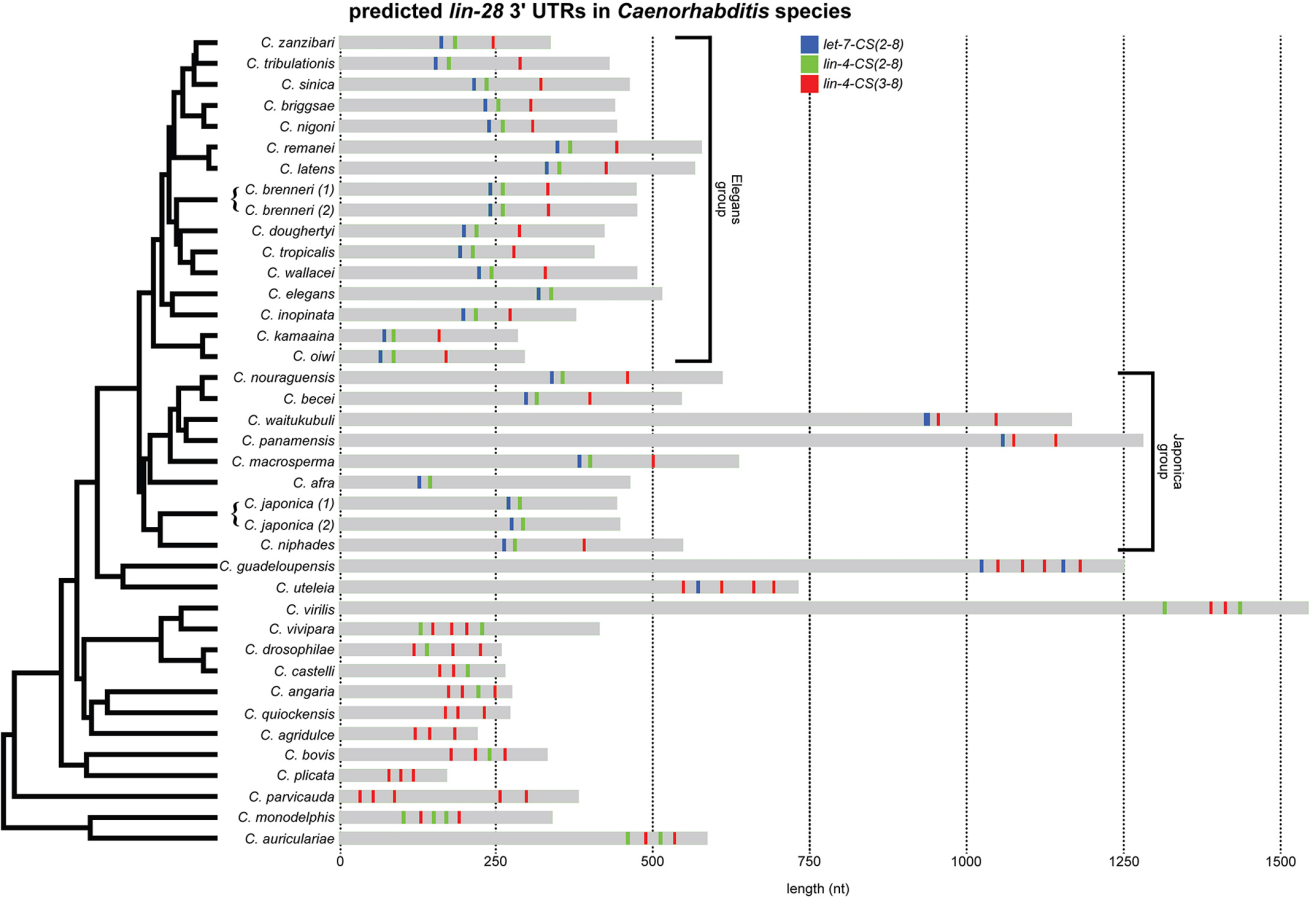
**Conservation of the *let-7* and *lin-4* microRNA-binding sites in the *lin-28* 3′ UTRs of *Caenorhabditis* species.** Predicted *let-7*-*CSs* and *lin-4-CSs* in the probable *lin-28* 3′ UTRs of *Caenorhabditis* species. Blue and green boxes denote the locations of *let-7*-*CSs* and *lin-4*-*CSs* that show predicted base pairing with nucleotides 2-8 of their corresponding microRNAs. Red boxes indicate the locations of *lin-4*-*CSs* predicted to base pair with nucleotides 3-8 of the *lin-4* microRNA.

To assess the conservation of *let-7* and *lin-4* microRNA targeting of LIN-28 mRNA across *Caenorhabditis* species, we identified homologs of *C. elegans lin-28* using BLAST searches and examined the downstream sequences (likely 3′ UTRs) for *let-7-CSs* and *lin-4-CSs*. All species analyzed possessed a single copy of *lin-28*, except for *C. brenneri* and *C. japonica*, which contained two copies (Fig. 1; Table S1).

All predicted *lin-28* 3′ UTR regions in the Elegans group contained a *let-7-CS* with complementarity to nucleotides 2-8 [hereafter referred to as *let-7*-*CS(2-8)*] followed by a *lin-4*-*CS(2-8)* (Fig. 1; Table S1). All 3′ UTR regions in Elegans group species, except for *C. elegans*, harbored a second *lin-4*-*CS* with complementarity to nucleotides 3-8 [*lin-4*-*CS(3-8)*] (Fig. 1; Table S1). In the Japonica group, all *lin-28* 3′ UTR regions exhibited a *let-7-CS(2-8)* followed by either a *lin-4-CS(2-8)* or a *lin-4-CS(3-8)* (Fig. 1; Table S1). Six of the nine Japonica group 3′ UTR regions also contained an additional *lin-4-CS(3-8)* (Fig. 1; Table S1). Outside of the Elegans and Japonica groups, only *C. guadeloupensis* and *C. uteleia* exhibited a *let-7-CS* and multiple *lin-4-CSs* in their *lin-28* 3′ UTR regions, while all other *lin-28* 3′ UTR regions exhibited multiple *lin-4-CSs* but no *let-7-CSs* (Fig. 1; Table S1).

### The *let-7* and *lin-4* microRNAs regulate LIN-28 expression redundantly

**Fig. 2.**
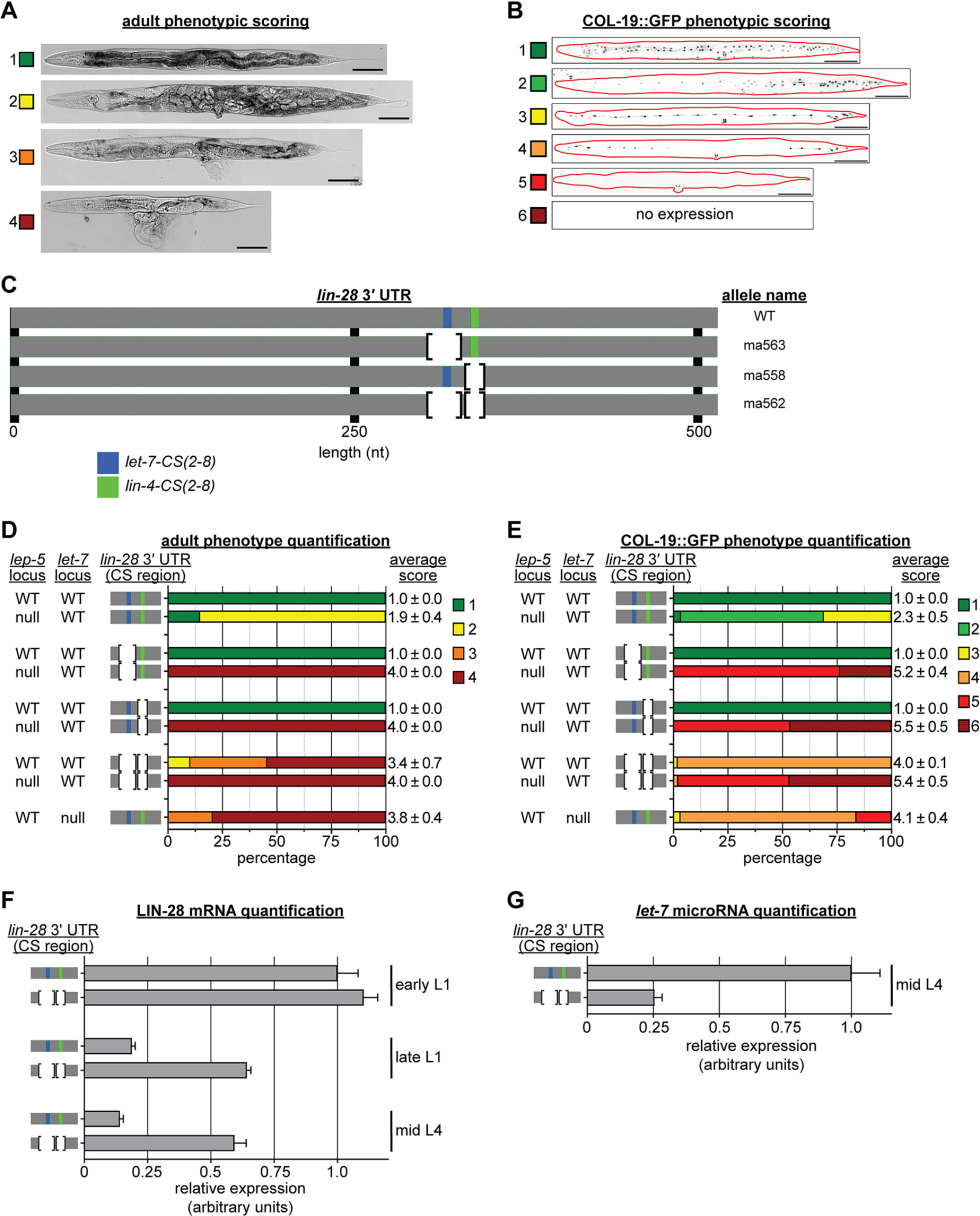
**The loss of *let-7* and *lin-4* microRNA targeting of LIN-28 mRNA results in retarded development, exacerbated by the removal of the *lep-5* lncRNA.** (A,B) Representative images of retarded developmental phenotypes impacting vulval function in adult *C. elegans* (A), the varying expression patterns of the collagen reporter *pCol-19::GFP* (B) and the associated scores for quantification. A description of the scoring methods can be found in the Materials and Methods section. Scale bars: 100 μm. (C) Depiction of the *C. elegans lin-28* 3′ UTR highlighting the *let-7-CS* and the *lin-4-CS* and the alleles used in this figure. (D,E) Quantification of adult (D) and COL-19::GFP (E) phenotypes of animals described in B with and without endogenous *lep-5* as well as *let-7(ma393)*. *n* (top to bottom)=44, 102, 58, 73, 60, 87, 79, 40 and 68 (D) and 42, 58, 72, 51, 88, 56, 46, 45 and 62 (E). Data are mean±s.d. (F,G) RT-qPCR analysis of LIN-28 mRNA (*n*=4) (F) and of *let-7* microRNA (*n*=5) (G) from whole animal extracts from wild-type and *lin-28(*Δ*CSs)*. Data are mean±s.d.

We hypothesized that loss of *let-7* and/or *lin-4* microRNA-mediated targeting of LIN-28 mRNA in *C. elegans* would lead to retarded developmental phenotypes due to misexpression of LIN-28. In *C. elegans*, such phenotypes often manifest as defects in vulval formation and integrity, as well as abnormal expression patterns of the adult-specific transgenic reporter *pCol-19::GFP* (Reinhart et al., 2000; Abbott et al., 2005)*.* To quantify the penetrance of retarded development, we devised two scoring methods for young adult specimens: (1) assessing the penetrance of vulval dysfunction (referred to as ‘adult phenotype’; Fig. 2A) and (2) evaluating the expression pattern of *pCol-19::GFP* (referred to as ‘COL-19::GFP phenotype’; Fig. 2B).

To examine the effects of *let-7* and *lin-4* microRNA regulation on *C. elegans* LIN-28 expression, we employed CRISPR/Cas9 to delete the *let-7-CS(2-8)* and *lin-4-CS(2-8)* (hereafter referred to as the *let-7-CS* and *lin-4-CS*) located within the 3′ UTR of the endogenous *lin-28* locus. Single-site deletions of either the *let-7-CS* [*lin-28(ma563)*, hereafter referred to as *lin-28(*Δ*let-7-CS)*; Fig. 2C] or the *lin-4-CS* [*lin-28(ma558)*, hereafter referred to as *lin-28(*Δ*lin-4-CS)*; Fig. 2C] did not result in observable phenotypes (Fig. 2D,E). However, concurrent deletion of both CSs [*lin-28(ma562)*, hereafter referred to as *lin-28(*Δ*CSs)*; Fig. 2C] caused retarded developmental phenotypes, indicating that the *let-7-CS* and *lin-4-CS* redundantly negatively regulate LIN-28 expression (Fig. 2D,E).

To investigate whether *lin-28* targeting by *let-7* and *lin-4* microRNAs destabilizes its mRNA, we quantified LIN-28 mRNA using reverse transcription quantitative polymerase chain reaction (RT-qPCR) in wild-type and *lin-28(*Δ*CSs)* mutants at the early L1-stage (before *lin-4* or let-7 family expression), late L1-stage (following *lin-4* expression) and mid L4-stage (following let-7 family expression). We observed no significant differences in LIN-28 mRNA levels in early L1 samples but observed an ∼3-fold increase in *lin-28(*Δ*CSs)* animals compared to wild-type at late L1 and mid L4 stages (Fig. 2F), indicating that *lin-4* and *let-7* microRNAs contribute to LIN-28 mRNA destabilization.

In *C. elegans*, *let-7* microRNA levels peak during the L4 stage (Reinhart et al., 2000). Its expression at earlier stages is inhibited by LIN-28, and *lin-28* null [*lin-28(0)*] mutants undergo precocious development, partly due to abnormally high *let-7* microRNA levels (Ambros and Horvitz, 1984; Johnson et al., 2003; Vadla et al., 2012). We hypothesized that misexpression of LIN-28 during later larval stages in *lin-28(*Δ*CSs)* animals would reduce *let-7* microRNA abundance compared to wild type. RT-qPCR showed that *let-7* microRNA levels in L4-stage larvae were ∼4-fold lower in *lin-28(*Δ*CSs)* than in wild-type, indicating that misexpression of LIN-28 in *lin-28(*Δ*CSs)* represses *let-7* microRNA expression (Fig. 2G). These results suggest that reduced *let-7* microRNA likely contributes to the retarded phenotypes observed in this mutant.

### The *let-7* and *lin-4* microRNAs collaborate with the *lep-5* lncRNA to downregulate LIN-28

We hypothesized that if the *let-7* and *lin-4* microRNAs were the sole negative regulators of LIN-28 expression, the phenotypes of *lin-28(*Δ*CSs)* animals would mimic those of *let-7* null [*let-7(0)*] animals. However, *lin-28(*Δ*CSs)* animals exhibited milder developmental delays compared to *let-7(0)* animals (Fig. 2D,E), suggesting that an additional negative regulatory mechanism contributes to LIN-28 repression in later larval stages. The *lep-5* lncRNA has been proposed to act in later larval stages to remove residual LIN-28 that was translated before the expression of the *let-7* and *lin-4* microRNAs (Kiontke et al., 2019). We postulated that the *lep-5* lncRNA may also promote the degradation of nascent LIN-28 protein translated at later larval stages, functioning with the *let-7* and *lin-4* microRNAs to maintain low LIN-28 expression.

To investigate this, we used CRISPR/Cas9 to delete the *lep-5* locus from the *C. elegans* genome [*lep-5(ma613)*; hereafter referred to as *lep-5(0)*]. Consistent with previous findings, *lep-5(0)* animals exhibited mild developmental retardation (Fig. 2D,E) (Kiontke et al., 2019). However, *lin-28(*Δ*CSs)*; *lep-5(0)* double mutants exhibited more pronounced developmental retardation than either single mutant, displaying phenotypes slightly more severe than *let-7(0)* mutants (Fig. 2D,E). Moreover, both *lin-28(*Δ*let-7-CS)*; *lep-5(0)* and *lin-28(*Δ*lin-4-CS)*; *lep-5(0)* double mutants exhibited similarly profound developmental retardation, mirroring *lin-28(*Δ*CSs)*; *lep-5(0)* double mutants (Fig. 2D,E), indicating that the removal of either CS sensitizes *C. elegans* to further perturbations in LIN-28 expression.

Similar to *let-7(0)* mutants, which are lethal at the L4 molt and require balancing with a wild-type copy of *let-7* for propagation, deletion of *lep-5* from *lin-28(*Δ*let-7-CS)*, *lin-28(*Δ*lin-4-CS)* or *lin-28(*Δ*CSs)* exhibits similar adult lethality. Homozygous double mutants must be balanced with a transgenic copy of *lep-5* on an extrachromosomal array (*maEx268* or *maEx269*). As is typical of extrachromosomal arrays, *maEx268* and *maEx269* exhibit meiotic instability, allowing us to assess the phenotypes of non-array-containing self-progeny of array-bearing hermaphrodites.

### The *let-7* and *lin-4* microRNAs, along with the *lep-5* lncRNA, are the main negative regulators of LIN-28 expression in seam and vulval cells

Loss of *lin-28* results in precocious expression of seam and vulva cell fates normally associated with later developmental stages (Ambros and Horvitz, 1984; Euling and Ambros, 1996). In contrast, retarded development of seam and vulva cell fates are observed in *lin-28(*Δ*CSs)* and *lep-5(0)* mutants (Fig. 2D,E). This indicates that the normal progression from larval to adult cell fates in these lineages relies on the downregulation of LIN-28 throughout development.

**Fig. 3.**
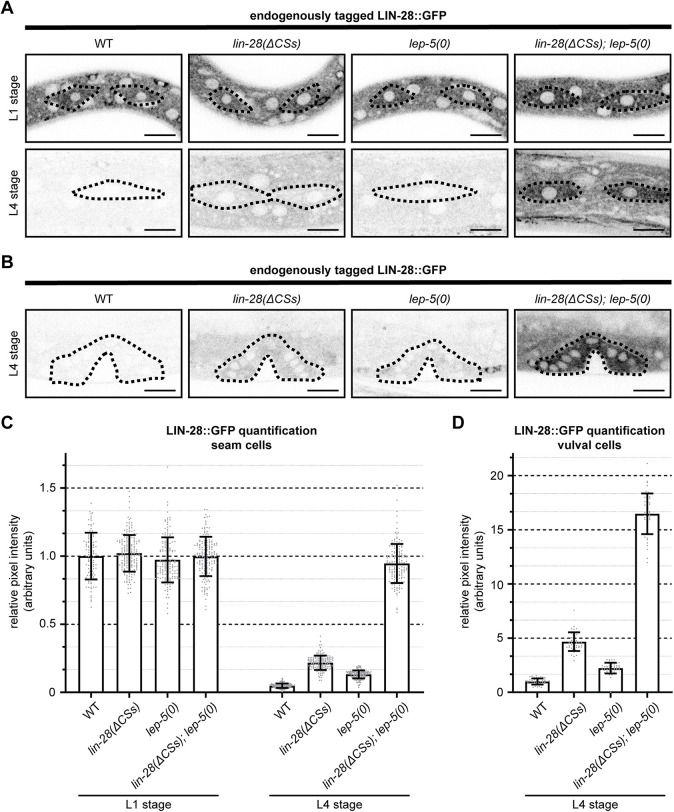
**The *let-7* and *lin-4* microRNAs negatively regulate LIN-28 protein expression synergistically with *lep-5* lncRNA.** (A-D) Representative images of endogenously tagged LIN-28::GFP in seam cells (A) and vulva cells (B) in wild type (wt), *lin-28(*Δ*CSs)*, *lep-5(0)* and *lin-28(*Δ*CSs)*; *lep-5(0)*, and their quantifications (C,D). Representative cells are outlined in black. *n* (left to right)=108, 186, 156, 171, 147, 147, 165 and 153 (C); 46, 45, 54 and 47 (D). Data are mean±s.d. Scale bars: 10 μm.

To assess the contributions of the *lin-4* and *let-7* microRNAs and the *lep-5* lncRNA to LIN-28 expression dynamics in seam and vulva cells, we quantified LIN-28 levels using endogenously tagged *lin-28::GFP* (Ilbay et al., 2021). We found that LIN-28::GFP expression in seam cells of wild-type animals was highest during the L1 larval stage and significantly decreased by the L4 stage (Fig. 3A,C). Developing vulval cells in L4 animals showed minimal LIN-28::GFP (vulval cells are not produced until after the L1 stage, precluding direct comparison to L4 vulval cells; Fig. 3B,D).

We next used CRISPR/Cas9 to generate the *lin-28(*Δ*CSs)* allele in *lin-28::GFP*-tagged animals. At the L1 stage, all mutants examined [*lin-28::GFP(*Δ*CSs)*, *lep-5(0)* and *lin-28::GFP(*Δ*CSs)*; *lep-5(0)*] exhibited nearly identical LIN-28::GFP expression in seam cells compared to wild-type (Fig. 3A,C). At the L4 stage, *lin-28::GFP(*Δ*CSs)* animals showed ∼4.5-fold and 4.7-fold increases in LIN-28::GFP expression in seam cells and vulval cells, respectively, relative to wild-type (Fig. 3C,D). *lep-5(0)* animals displayed ∼2.7-fold and 2.2-fold respective increases in LIN-28::GFP expression in seam cells and vulval cells at the L4 stage (Fig. 3C,D). Strikingly, at the L4 stage, *lin-28::GFP(*Δ*CSs)*; *lep-5(0)* double mutants exhibited ∼19.4-fold and 16.5-fold respective de-repression of LIN-28::GFP in seam cells and vulval cells (Fig. 3C,D).

### GFP tagging of LIN-28 reduces the phenotypic effects of *lin-28* gain-of-function mutations

As described above, *lin-28(*Δ*CSs)* animals exhibit gain-of-function phenotypes that result in retarded development (Fig. 2D,E). However, *lin-28::GFP(*Δ*CSs)* animals did not display any apparent retarded developmental phenotypes (Fig. S1A), suggesting that the GFP tag on LIN-28 may alter its developmental dynamics and/or reduce LIN-28 protein activity. To investigate this, we employed RT-qPCR to measure LIN-28 mRNA levels and western blotting with an anti-LIN-28 antibody to assess LIN-28 protein levels. The results indicated a subtle reduction in LIN-28::GFP mRNA during the early-L1 stage compared to untagged LIN-28 mRNA; however, no significant differences were observed in late-L1 and mid-L4 stages (Fig. S1B). We found no significant differences between untagged and GFP-tagged LIN-28 protein levels in L1 and L4 samples (Fig. S1C).

These findings indicate that the suppression of gain-of-function phenotypes in *lin-28::GFP(*Δ*CSs)* animals results from a hindrance of LIN-28 function caused by the GFP tag. To assess the reduction in LIN-28 activity conferred by the GFP tag, we examined *lin-28::GFP* animals for phenotypic hallmarks of *lin-28* loss of function. They did not exhibit any discernible loss-of-function phenotypes and appeared to be indistinguishable from wild type (Fig. S1A), indicating that the GFP tag does not sufficiently hinder LIN-28 function to cause a phenocritical degree of loss of function.

To assess any cryptic *lin-28* loss of function associated with *lin-28::GFP*, we measured *let-7* microRNA levels in L4 larvae using RT-qPCR. Elevated *let-7* microRNA levels indicate reduced LIN-28 activity, as *let-7* microRNA levels are negatively regulated by LIN-28. We found that *let-7* microRNA levels were ∼1.6-fold greater in *lin-28::GFP* animals compared to untagged counterparts, suggesting a slight reduction in LIN-28 function with GFP tagging (Fig. S1D).

These findings suggest that the GFP tag on LIN-28::GFP subtly reduces LIN-28 activity enough to suppress *lin-28* gain-of-function phenotypes. Notably, LIN-28 mRNA levels increased similarly with CS deletions, regardless of the GFP tag (Fig. S1E). Moreover, the *lin-28::GFP(*Δ*CSs)*; *lep-5(0)* double mutant exhibited retarded phenotypes indistinguishable from the untagged counterpart, *lin-28:(*Δ*CSs)*; *lep-5(0)* (Fig. S1A), indicating that the net effect of *lin-28::GFP*(ΔCSs*)* is *lin-28* gain of function.

### The 3′ UTR of *lin-28* is non-essential under standard laboratory conditions and contains several positive regulatory elements

**Fig. 4.**
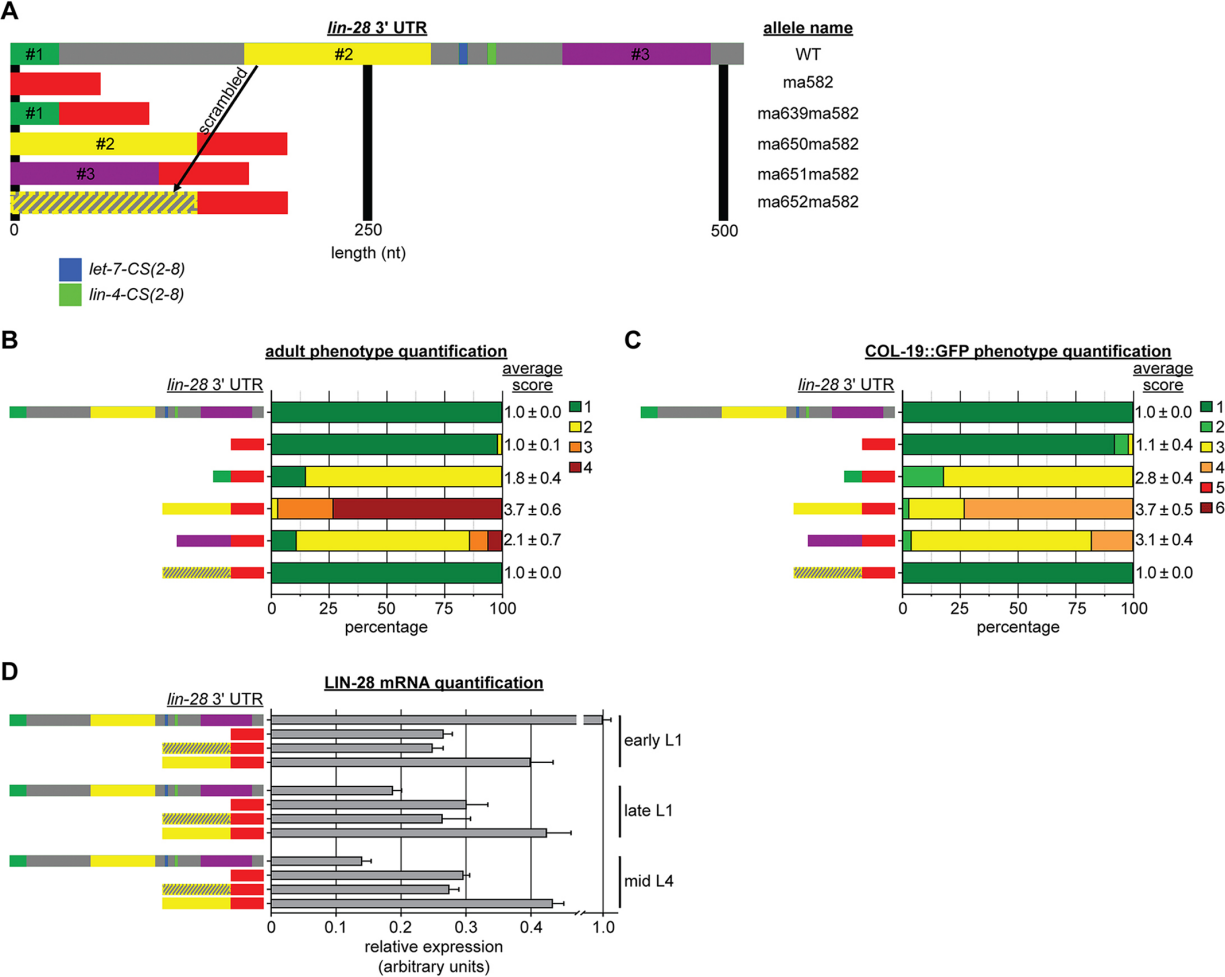
**The *lin-28* 3′ UTR contains multiple positive regulatory sequences.** (A) Depiction of the *lin-28* 3′ UTR alleles used for this figure. Note, *lin-28(ma652)* is a scrambled version of *ma650*. (B,C) Quantification of adult (B) and COL-19::GFP (C) phenotypes of animals described in A. Wild-type data are the same as Fig. 2D. *n* (from top to bottom)=44, 54, 125, 55, 96 and 72 (B); 42, 51, 71, 55, 51 and 64 (C). Data are mean±s.d. (D) RT-qPCR analysis of LIN-28 mRNA from whole animal extracts from wild-type, *lin-28(ma582)*, *lin-28(ma652ma582)* and *lin-28(ma650ma582).* Wild-type data are the same as Fig. 2F. *n*=4. Data are mean±s.d.

To determine if the *let-7-CS* and *lin-4-CS* are the predominant regulatory sequences in the *lin-28* 3′ UTR, we employed CRISPR/Cas9 to delete the entire *lin-28* 3′ UTR and replace it with a 64-bp segment of the *act-1* 3′ UTR [*lin-28(ma582)*; hereafter referred to as *lin-28(*Δ*3′ UTR)*; Fig. 4A]. Unexpectedly, *lin-28(*Δ*3′ UTR)* animals appeared to be phenotypically wild-type despite lacking the *let-7-CS* and *lin-4-CS* (Fig. 4B,C), suggesting that the *lin-28* 3′ UTR contains sequences outside the negative regulatory *let-7-CS* and *lin-4-CS* that have a positive regulatory effect.

To investigate whether positive regulatory elements contribute to the misexpression of LIN-28 in the absence of the *let-7-CS* and *lin-4-CS*, we used CRISPR/Cas9 to generate strains with portions of the endogenous *lin-28* 3′ UTR deleted. We assessed these strains for retarded developmental phenotypes (Fig. S2A,B) and identified three regions within the *lin-28* 3′ UTR that likely possess positive regulatory functions (referred to as positive element #1, positive element #2 and positive element #3; Fig. S2B,C). Notably, all 3′ UTR truncation mutants that retained the CSs exhibited wild-type phenotypes, suggesting that *let-7* and *lin-4*-mediated repression of LIN-28 does not require an intact 3′ UTR and can counteract any positive effect of LIN-28 expression conferred by other sequences within the 3′ UTR (Fig. S2B).

Alignment and motif analyses of previously identified *Caenorhabditis* species *lin-28* 3′ UTR homologs revealed conserved sequences in positive elements #2 and #3, but not in #1 (Fig. S3; Table S2). These results suggest conservation of positive regulatory roles of sequences in *lin-28* 3′ UTR (positive elements #2 and #3) and *C. elegans*-specific regulation of LIN-28 expression (positive element #1).

To test if these positive regulatory elements could promote LIN-28 misexpression without residual endogenous *lin-28* 3′ UTR sequences, we used CRISPR/Cas9 to insert each region at the beginning of the artificial 3′ UTR of *lin-28(*Δ*3′ UTR)* animals and assessed them for phenotypes indicative of LIN-28 misexpression [*lin-28(ma639ma582)*, *lin-28(ma650ma582)*, *lin-28(ma651ma582)* and *lin-28(ma652ma582)*; Fig. 4A]. Each region caused varying degrees of developmental retardation compared to a scrambled control, which exhibited characteristics indistinguishable from wild-type (Fig. 4B,C). These findings indicate that each element is sufficient to promote LIN-28 expression without the presence of the rest of the *lin-28* 3′ UTR.

*lin-28(*Δ*CSs)* animals exhibit elevated levels of LIN-28 mRNA at later stages compared to wild-type animals, likely due to reduced microRNA-mediated mRNA turnover (Fig. 2F). Although *lin-28(*Δ*3*′ *UTR)* animals display a wild-type phenotype, they lack the *let-7-CS* and *lin-4*-*CS* and could be expected to show impaired downregulation of LIN-28 mRNA compared to wild type. Using RT-qPCR, we quantified LIN-28 mRNA levels in *lin-28(*Δ*3*′ *UTR)* animals at early L1, late L1 and mid L4 larval stages and observed similar levels of LIN-28 mRNA at all time points, consistent with an absence of 3′ UTR-mediated downregulation of LIN-28 mRNA in *lin-28(*Δ*3*′ *UTR)* animals (Fig. 4D). However, the levels of LIN-28 mRNA in *lin-28(*Δ*3*′ *UTR)* mutants were ∼25% of the levels observed in early L1-stage wild-type animals (Fig. 4D). This indicates that *lin-28* 3′ UTR sequences outside of the *let-7-CS* and *lin-4*-*CS* promote LIN-28 mRNA accumulation at all larval stages.

Decreased LIN-28 mRNA levels in *lin-28(*Δ*3*′ *UTR)* mutants led us to hypothesize that positive elements in the 3′ UTR enhance LIN-28 expression by stabilizing its mRNA. Using RT-qPCR, we measured LIN-28 mRNA levels at early L1, late L1 and mid L4 larval stages in the strain with the most potent positive element inserted into the artificial 3′ UTR of *lin-28(*Δ*3′ UTR)* animals [*lin-28(ma650ma582)*; hereafter referred to as *lin-28(positive #2::*Δ*3′ UTR)*; Fig. 4A]. In these *lin-28(positive #2::*Δ*3′ UTR)* animals, mRNA levels remained relatively constant throughout development, representing ∼40% of wild-type early L1 levels and approximately one-third higher than those in *lin-28(*Δ*3′ UTR)* animals (Fig. 4D), supporting the conclusion that positive elements within the *lin-28* 3′ UTR facilitate LIN-28 expression by stabilizing its mRNA.

### The 3′ UTR-mediated regulation of *lin-28* functions independently of *lep-5*-mediated post-translational regulation

**Fig. 5.**
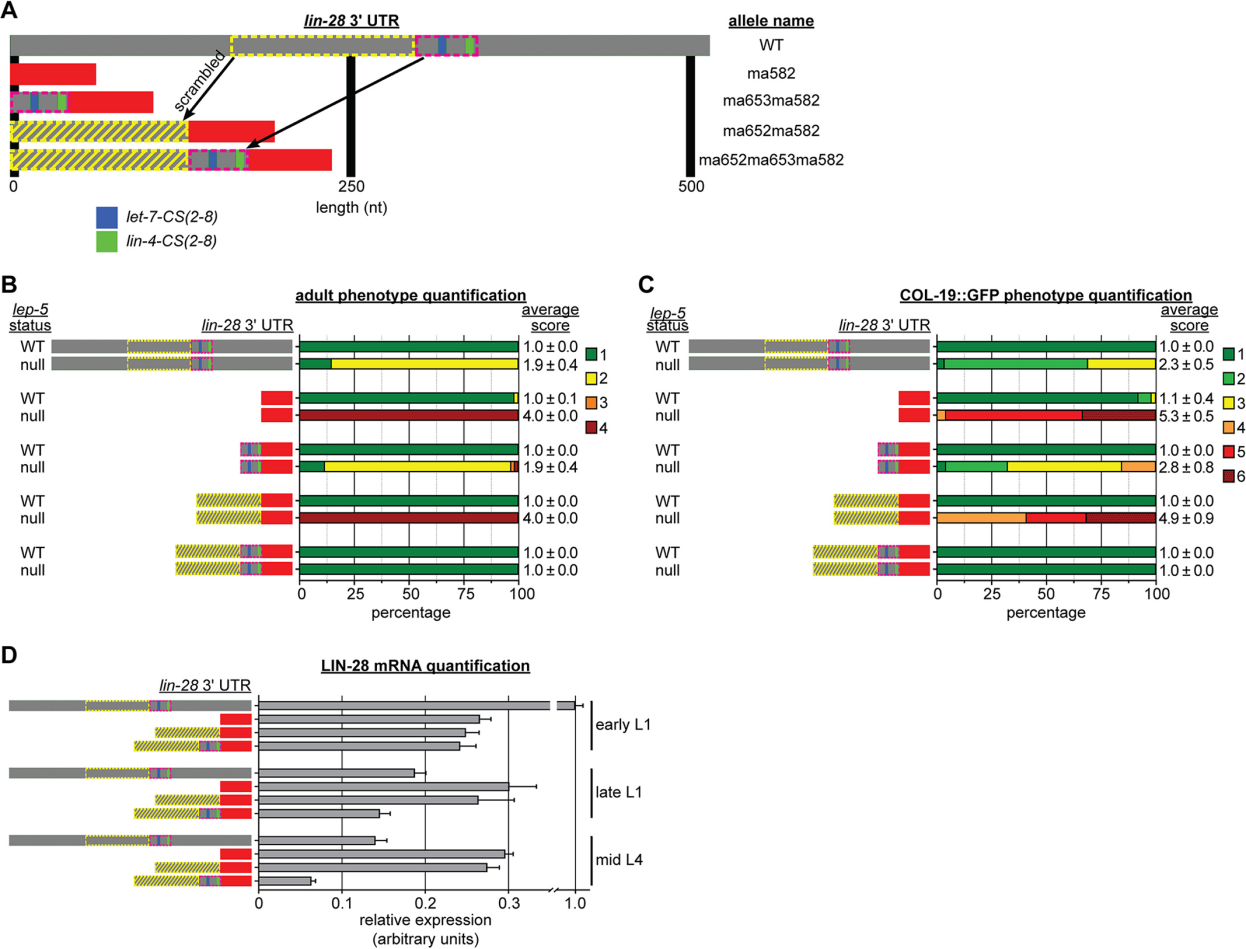
**The *lin-28* 3′ UTR is non-essential under standard conditions.** (A) Depiction of the *lin-28* 3′ UTR alleles used for this figure. (B,C) Quantification of adult (B) and COL-19::GFP (C) phenotypes of animals described in A with and without endogenous *lep-5*. Wild-type data are the same as Fig. 2D. *lin-28(ma582)* and *lin-28(ma652ma582)* data with wild-type *lep-5* are the same as Fig. 4B. *n* (from top to bottom)=44, 102, 54, 43, 142, 120, 72, 62, 98 and 142 (B); 42, 58, 51, 48, 60, 71, 64, 95, 84 and 82 (C). Data are mean±s.d. (D) RT-qPCR analysis of LIN-28 mRNA from whole animal extracts from wild-type, *lin-28(ma582)*, *lin-28(ma652ma582)* and *lin-28(ma652ma653ma582).* Wild-type 3′ UTR data are the same as Fig. 2F. *lin-28(ma582)* and *lin-28(ma652ma582)* data are the same as Fig. 4D. *n*=4. Data are mean±s.d.

The essentially wild-type phenotype of *lin-28(*Δ*3′ UTR)* animals indicated that LIN-28 is downregulated by a 3′ UTR-independent mechanism. *lin-28(*Δ*3′ UTR)*; *lep-5(0)* double mutants showed severe developmental retardation indistinguishable from *lin-28(*Δ*CSs)*; *lep-5(0)* double mutants (Fig. 5A-C), indicating that *lep-5* is responsible for LIN-28 downregulation in the absence of the 3′ UTR. Therefore, normal LIN-28 dynamics involve both 3′ UTR-mediated regulation of LIN-28 synthesis and 3′ UTR-independent regulation of LIN-28 stability by *lep-5* lncRNA.

### LIN-28 gain of function leads to increased seam cell numbers

In wild-type animals, a significant portion of LIN-28 downregulation occurs during the L2 stage. Loss of *lin-28* results in skipping L2-specific seam cell divisions and fewer adult seam cells (Ambros and Horvitz, 1984). We hypothesized that gain of function of LIN-28 could increase seam cell numbers due to reiteration of L2 divisions. To test this, we crossed the pScm::GFP seam cell reporter into *lin-28(*Δ*CSs)*, *lin-28(*Δ*3′ UTR)*, *lep-5(0)* and respective double mutants. Seam cell counts in *lin-28(*Δ*3′ UTR)* and *lep-5(0)* single mutants were nearly indistinguishable from wild-type, whereas seam cell numbers were significantly elevated in the *lin-28(*Δ*CSs)* single mutant and in *lin-28(*Δ*CSs)*; *lep-5(0)* and *lin-28(*Δ*3′ UTR)*; *lep-5(0)* double mutants during L3, L4 and young adult stages (Fig. S4). These findings suggest that significant misexpression of LIN-28 leads to reiteration of L2-stage seam cell fates at later larval stages.

### The *let-7-CS* and *lin-4-CS* can function independently of the rest of the *lin-28* 3′ UTR

The severe LIN-28 misexpression phenotypes of *lin-28(*Δ*3′ UTR)*; *lep-5(0)* double mutants allowed us to test whether the *let-7-CS* and *lin-4-CS* can function without other 3′ UTR sequences. We used CRISPR/Cas9 to insert a 42-bp sequence derived from the wild-type *lin-28* 3′ UTR, including both the *let-7-CS* and the *lin-4-CS*, at the beginning of the artificial 3′ UTR in *lin-28(*Δ*3′ UTR)* animals [*lin-28(ma653ma582)*; hereafter referred to as *lin-28(CSs::*Δ*3*′ *UTR)*; Fig. 5A] and investigated whether this sequence could suppress the severely retarded developmental phenotypes of *lin-28(*Δ*3′ UTR)*; *lep-5(0)* double mutant animals. The *lin-28(CSs::*Δ*3*′ *UTR)*; *lep-5(0)* animals showed a significant but incomplete suppression of the *lin-28* gain-of-function phenotypes of *lin-28(*Δ*3*′ *UTR)*; *lep-5(0)* animals and did not require balancing with a transgenic *lep-5* array (Fig. 5B,C). These findings indicate that the *let-7-CS* and *lin-4-CS* segment of the *lin-28* 3′ UTR can downregulate LIN-28 independently of other 3′ UTR sequences.

The incomplete suppression of the *lin-28* gain-of-function phenotypes in *lin-28(CSs::*Δ*3*′ *UTR)*; *lep-5(0)* animals led us to hypothesize that the sequence containing the CSs may not be optimally positioned for full microRNA activity due to its proximity to the *lin-28* coding region. We used CRISPR/Cas9 to insert the scrambled sequence used earlier (*ma652*; Fig. 4A) between the *lin-28* stop codon and the CSs of *lin-28(CSs::*Δ*3*′ *UTR)* animals [*lin-28(ma652ma653ma582)*; henceforth referred to as *lin-28(spacer::CSs::*Δ*3*′ *UTR)*; Fig. 5A]. Insertion of the spacer upstream of the CSs [*lin-28(spacer::CSs::*Δ*3*′ *UTR)*; *lep-5(0)*] resulted in complete suppression of the *lin-28* gain-of-function phenotypes observed in *lin-28(*Δ*3*′ *UTR)*; *lep-5(0)* animals, reinforcing the conclusion that the *let-7-CS* and *lin-4-CS* can function independently of other native *lin-28* 3′ UTR sequences, and that positioning the CSs further from the *lin-28* stop codon is more optimal for *let-7* and *lin-4-*mediated repression of LIN-28 expression (Fig. 5B,C). The insertion of the spacer without the CSs did not suppress the gain-of-function phenotypes of *lin-28(*Δ*3*′ *UTR)*; *lep-5(0)* [*lin-28(spacer::*Δ*3*′ *UTR*; *lep-5(0)*; Fig. 5B,C].

In the absence of other *lin-28* 3′ UTR sequences, especially the positive elements, we expected that restoring *let-7* and *lin-4* microRNA repression in the *lin-28(spacer::CSs::*Δ*3*′ *UTR)* construct might result in precocious phenotypes characteristic of *lin-28* loss of function. However, *lin-28(spacer::CSs::*Δ*3′ UTR)* animals did not exhibit any discernible loss-of-function phenotypes and appeared phenotypically indistinguishable from wild-type (Fig. 5B,C).

Based on our findings (Fig. 2F), we hypothesized that the addition of CSs in *lin-28(spacer::CSs::*Δ*3*′ *UTR)* animals would destabilize LIN-28 mRNA at later larval stages. RT-qPCR showed decreased LIN-28 mRNA levels in late L1 and mid L4 samples compared to wild-type and controls lacking CSs (Fig. 5D). This suggests that *let-7* and *lin-4* microRNAs repress LIN-28 expression by destabilizing LIN-28 mRNA independently of the rest of the *lin-28* 3′ UTR.

### The deletion of the entire *lin-28* 3′ UTR leads to mild but significant alterations in the developmental dynamics of LIN-28 expression

**Fig. 6.**
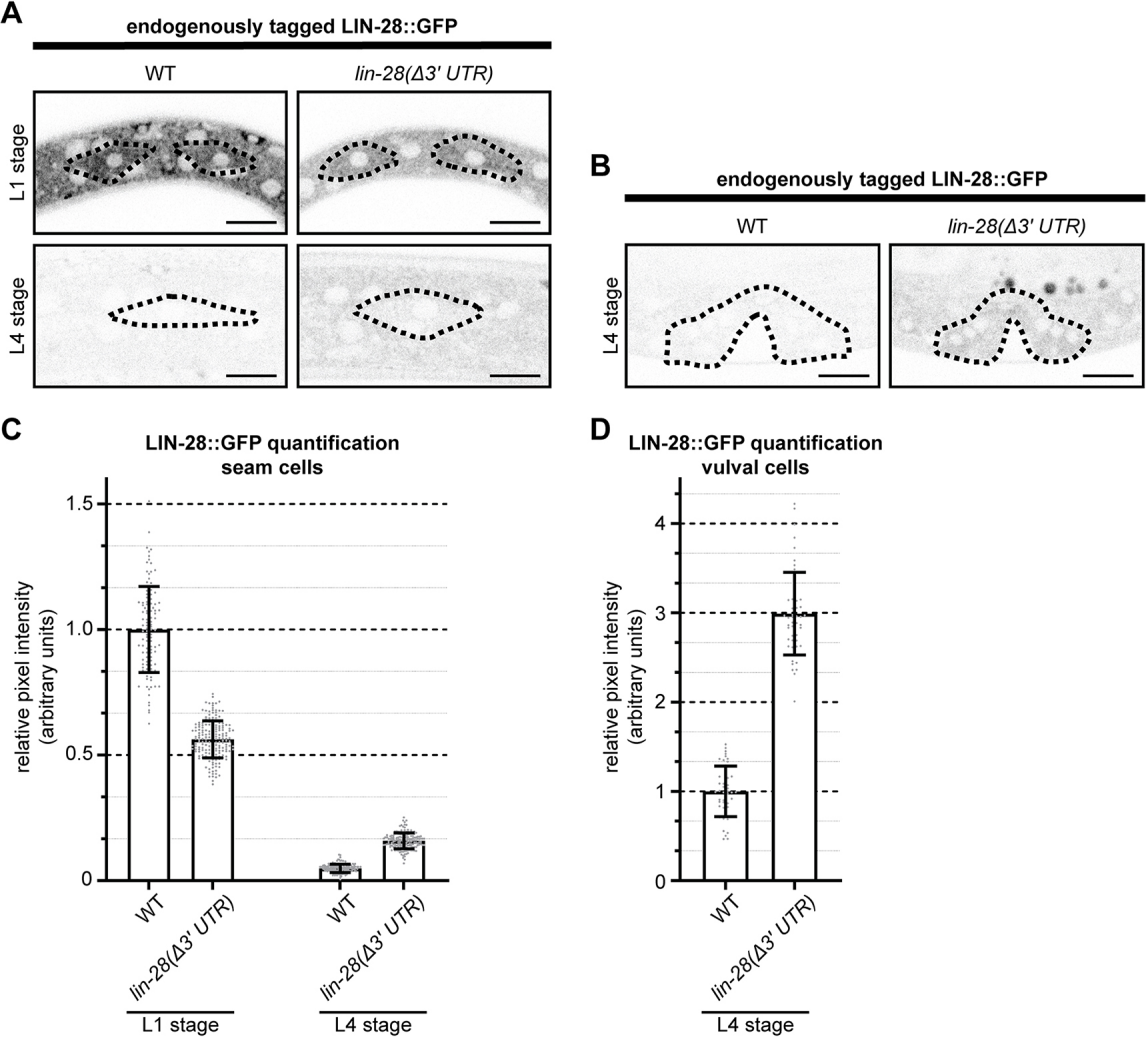
**The 3′ UTR of *lin-28* enhances LIN-28::GFP expression during early development.** (A-D) Representative images of endogenously tagged LIN-28::GFP in seam cells (A) and vulva cells (B) in wild-type and *lin-28(*Δ*3′ UTR)* and their respective quantifications (C,D). Wild-type images and data are the same as Fig. 3A-D. Representative cells are outlined in black. *n* (from left to right)=108, 183, 165 and 165 (C); 46 and 52 (D). Scale bars: 10 μm.

Prompted by our finding that LIN-28 mRNA from *lin-28(*Δ*3*′ *UTR)* animals was decreased in L1 larvae and elevated in L4 larvae compared to wild type (Fig. 4D), we used CRISPR/Cas9 to generate *lin-28::GFP(*Δ*3*′ *UTR)* animals to test whether LIN-28 protein levels would show correspondingly disrupted developmental dynamics in the absence of the *lin-28* 3*′* UTR. We observed that LIN-28::GFP expression in *lin-28::GFP(*Δ*3*′ *UTR)* animals was ∼50% lower in L1-stage seam cells and ∼3-fold higher in both L4-stage seam and vulval cells compared to wild type (Fig. 6A-D). This supports the model that the *lin-28* 3′ UTR mediates both positive and negative regulatory inputs, promoting LIN-28 expression during early developmental stages and repressing expression in later stages.

### The heterochronic phenotypes of *lin-28* 3′ UTR mutants exhibit sensitivity to development in the presence of the pathogen *Pseudomonas aeruginosa* or at specific temperatures

In *C. elegans*, let-7 family microRNAs miR-48/84/241 are abundantly expressed in the L2 and L3 stages, while *let-7* microRNA is upregulated in the L4 stage. Thus, much of the let-7 family-mediated downregulation of LIN-28 results from the combined action of miR-48/84/241. Loss-of-function mutants of *mir-48/84/241* exhibit retarded heterochronic phenotypes analogous to LIN-28 misexpression mutants (Abbott et al., 2005).

The developmental phenotypes of let-7 family mutants can be influenced by environmental conditions. For example, exposure of *mir-48/241* compound mutants to the pathogenic bacterium *Pseudomonas aeruginosa* exacerbates their developmental timing phenotypes in an innate immunity-dependent manner, demonstrating that these microRNAs are relatively more crucial for specifying temporal cell fates during development under pathogen stress than during unstressed development (Ren and Ambros, 2015).

**Fig. 7.**
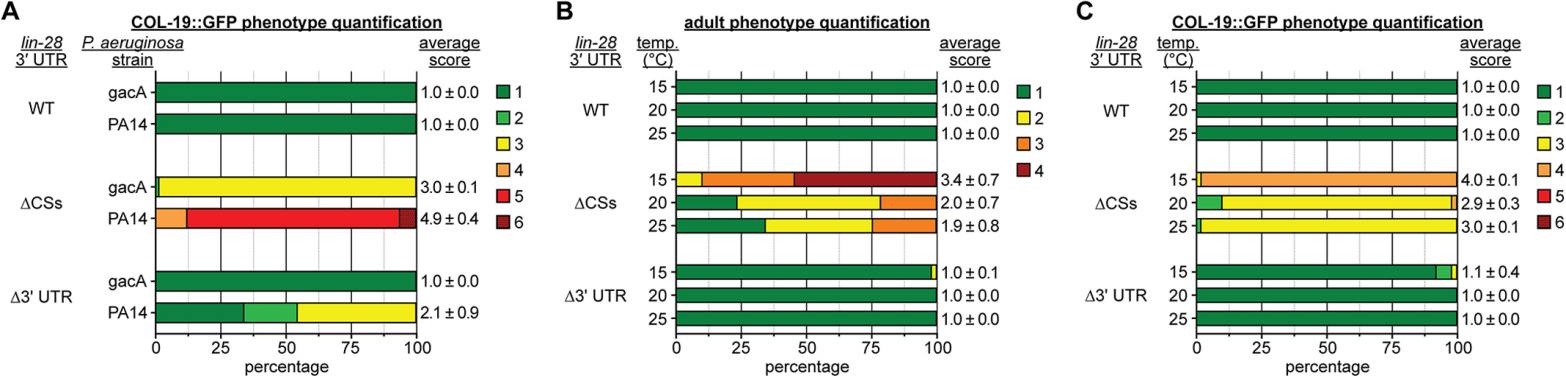
***lin-28* 3′ UTR mutants exhibit sensitivity to environmental stress.** (A-C) Quantification of COL-19::GFP (A,C) and adult (B) phenotypes of animals exposed to *P. aeruginosa* strains (A) and development at various temperatures (B,C). The 15°C wild-type and *lin-28(*Δ*CSs)* data are the same as Fig. 2D,E (B,C). The 15°C *lin-28(*Δ*3′ UTR)* data are the same as Fig. 4B,C. *n* (from top to bottom)=98, 44, 66, 49, 107 and 44 (A); 44, 63, 41, 79, 140, 90, 54, 69 and 62 (B); 42, 63, 53, 46, 50, 52, 51, 47 and 62 (C). Data are mean±s.d.

To test if the developmental timing phenotypes of *lin-28* 3′ UTR mutants are similarly sensitive to *P. aeruginosa*, we cultured wild-type, *lin-28(*Δ*CSs)* and *lin-28(*Δ*3′ UTR)* larvae on the pathogenic *P. aeruginosa* strain PA14 and the non-pathogenic GacA mutant of PA14 and assessed *pCol-19::GFP* expression by scoring young adults. *C. elegans* larvae and young adults tolerate PA14 as a food source; however, exposure to PA14 is lethal to adults, preventing full assessment of adult morphological phenotypes (Tan et al., 1999; Ren and Ambros, 2015; Mirza et al., 2023). Wild-type animals showed no differences in *pCol-19::GFP* expression when exposed to either PA14 or GacA (Fig. 7A). In contrast, the retarded developmental *pCol-19::GFP* phenotypes of *lin-28(*Δ*CSs)* animals were exacerbated upon exposure to PA14 compared to GacA (Fig. 7A). Similarly, *lin-28(*Δ*3′ UTR)* animals exhibited retarded *pCol-19::GFP* expression patterns when exposed to PA14 that were not evident with exposure to GacA (Fig. 7A). This finding indicates that misregulation of LIN-28 sensitizes larval cell fate progression to stressors associated with exposure to virulent *P. aeruginosa*.

Our previous experiments quantifying developmental phenotypes were conducted at 15°C, while exposure to PA14 required 25°C. We found that the developmental phenotypes of *lin-28(*Δ*CSs)* animals raised on GacA at 25°C were less severe than those at 15°C. To test temperature sensitivity, we raised wild-type, *lin-28(*Δ*CSs)* and *lin-28(*Δ*3′ UTR)* on *Escherichia coli* at 15°C, 20°C and 25°C. Wild-type and *lin-28(*Δ*3′ UTR)* animals showed no significant differences, while *lin-28(*Δ*CSs)* animals exhibited more pronounced retarded developmental phenotypes at 15°C compared to 20°C and 25°C (Fig. 7B,C).

The developmental rate of *C. elegans* at 15°C or when exposed to PA14 is slower than at 25°C or when exposed to non-pathogenic bacteria (Fig. 7A-C) (Nelson and Ambros, 2021; Mirza et al., 2023). To determine if the exacerbated phenotypes of *lin-28* 3′ UTR mutants cultured on *P. aeruginosa* are due to virulence or delayed development, we cultured wild-type, *lin-28(*Δ*CSs)* and *lin-28(*Δ*3′ UTR)* mutants on four additional *P. aeruginosa* strains that vary in their effects on developmental rate (referred to as DT50 for developmental time for 50% of animals to reach adulthood) and virulence (referred to as AMS for adult median survival) (Mirza et al., 2023).

Development of *lin-28(*Δ*CSs)* and *lin-28(*Δ*3′ UTR)* mutants on strains with similar virulence to PA14 (AZPAE14879, AZPAE14884 and MSH10) resulted in exacerbated retarded developmental phenotypes, while phenotypes on the mildly virulent strain PA01 were nearly identical to phenotypes on GacA (Fig. S5). Notably, the phenotypes of *lin-28(*Δ*CSs)* and *lin-28(*Δ*3′ UTR)* mutants exposed to strains that slowed development (AZPAE14884 and MSH10) were more severe than exposure to the similarly virulent strain AZPAE14879, which does not slow development (Fig. S5). These results indicate that both virulence and developmental rate slowing contribute to the exacerbated retarded developmental phenotypes of *lin-28(*Δ*CSs)* and *lin-28(*Δ*3′ UTR)* mutants when exposed to *P. aeruginosa.*

## DISCUSSION

In this study, we demonstrate that *lin-4* microRNA-mediated repression of LIN-28 is conserved among *Caenorhabditis* species, while *let-7* microRNA-mediated repression is mainly found in the Elegans and Japonica groups. We found that the *let-7-CS* and *lin-4-CS* in the *C. elegans lin-28* 3′ UTR semi-redundantly repress LIN-28 synergistically with the *lep-5* lncRNA. We revealed that the *lin-28* 3′ UTR contains cis-acting elements that positively regulate LIN-28 expression and counteract repression by the *let-7* and *lin-4* microRNAs. Additionally, we found that development of 3′ UTR mutants at varying culture temperatures or in the presence of the pathogenic bacterium *P. aeruginosa* can elicit cryptic heterochronic phenotypes.

In *C. elegans* and mammals, LIN-28 negatively regulates the *let-7* microRNA, so the loss of LIN-28 leads to increased *let-7* microRNA levels (Johnson et al., 2003; Viswanathan et al., 2008; Heo et al., 2008). The 3′ UTRs of LIN-28 mRNA in both *C. elegans* and mammals contain *let-7-CSs*, indicating a conserved negative regulatory relationship (Reinhart et al., 2000; Moss and Tang, 2003). However, in many of the *Caenorhabditis* species examined in this study, *lin-28* is predicted to be regulated by the *lin-4* microRNA but not by *let-7* microRNAs, despite the presence of *let-7* sequences in their genomes. This suggests that the *let-7*/*lin-28* mutual negative relationship, although broadly conserved, is not evolutionarily universal (Nelson and Ambros, 2021). Why many of these species appear to lack the otherwise highly conserved regulation of *lin-28* by *let-7* remains unclear and warrants further investigation.

Our analysis of *lin-28* 3′ UTR sequences among *Caenorhabditis* species revealed high conservation of both *lin-4-CS(2-8)* and *lin-4-CS(3-8)*. Notably, nucleotides 2-4 of the *lin-4* microRNA seed consist of a triplet of guanines, allowing for bulges at positions 2, 3 or 4 [still referred to as *lin-4-CS(3-8)*]. Our analysis indicated that the number of *lin-4-CS(3-8)* sites was greater than that of *lin-4-CS(2-8)* sites among analyzed *Caenorhabditis lin-28* 3′ UTRs (65 sites versus 35 sites, respectively). This pattern of conservation of seed pairing configurations suggests that the utilization of *lin-4-CS(3-8)* sites may confer distinct functional characteristics compared to *lin-4-CS(2-8)* sites.

We observed that deletion of either the *let-7-CS* or *lin-4-CS* from the *C. elegans lin-28* 3′ UTR resulted in wild-type phenotypes under standard conditions. However, removing the post-translational negative regulator *lep-5* lncRNA in these single-site deletion mutant backgrounds led to pronounced retarded developmental phenotypes, indicating extreme LIN-28 misexpression and semi-redundant function of these microRNA sites.

A previous study showed that the *lep-5* lncRNA is upregulated during the L2 stage and elicits post-translational degradation of LIN-28 (Kiontke et al., 2019). Our findings suggest a role for *lep-5* throughout L2-L4 development in degrading LIN-28 produced from ongoing LIN-28 mRNA translation. This is supported by the phenotypic interaction between *lin-28(*Δ*CSs)* and *lep-5(0)* mutations and the dramatically increased expression of LIN-28::GFP between the L1 and L4 stages in *lin-28(*Δ*CSs)*; *lep-5(0)* double mutants compared to single mutants. We conclude that the *lep-5* lncRNA functions continuously throughout the L2-L4 stages to attenuate LIN-28 accumulation, complementing microRNA-mediated inhibition of LIN-28 synthesis.

Previous investigations of LIN-28 repression during larval development found that at later stages, when LIN-28 protein levels are low, LIN-28 mRNA was associated with polyribosomes (Seggerson et al., 2002; Stadler et al., 2012). Our finding that the *lep-5* lncRNA downregulates LIN-28 during later larval stages by degrading newly synthesized LIN-28 explains these previous observations.

The phenotypic similarities between *lin-28* gain-of-function alleles and the *let-7* null allele, along with the negative regulation of *let-7* microRNA expression by LIN-28, suggest that reduced *let-7* microRNA levels contribute to developmental retardation in *lin-28* gain-of-function mutants. Notably, the phenotypes in *lin-28* 3′ UTR mutants lacking the *lep-5* lncRNA were more severe than those in *let-7(0)* mutants. This indicates that the phenotypes in these double mutants are likely due to the deregulation of targets of LIN-28 and/or *lep-5* lncRNA, in addition to *let-7* microRNA.

MicroRNAs modulate protein synthesis from target mRNAs by repressing mRNA stability and/or inhibiting translational efficiency (Cottrell et al., 2017). In the context of regulating *lin-28* by the *let-7* and *lin-4* microRNAs, our findings suggest the involvement of both mechanisms but do not conclusively elucidate their contributions. Quantification of LIN-28::GFP upregulation in seam cells at the L4 stage of *lep-5(0)* animals, with and without the CSs in the *lin-28* 3′ UTR, indicated an ∼7-fold effect of the *let-7* and *lin-4* microRNAs on LIN-28 levels. At the whole-animal level, LIN-28 mRNA expression is upregulated by ∼4-fold upon deletion of the CSs. Since the *lep-5* lncRNA does not affect LIN-28 mRNA levels (Kiontke et al., 2019), this 4-fold increase can be interpreted as mediated by microRNAs. A limitation of these interpretations lies in comparing whole-animal mRNA measurements to protein measurements in seam cells, as regulatory mechanisms may differ among distinct cell types. Furthermore, the impact of microRNAs on LIN-28 mRNA levels may be complicated by potential autoregulatory effects of LIN-28 protein on LIN-28 mRNA (Stefani et al., 2015).

*C. elegans* lacking the endogenous *lin-28* 3′ UTR appear to be wild-type despite lacking *let-7* and *lin-4* microRNA regulation. In contrast, the *lin-28(*Δ*CSs)* mutant displays retarded developmental phenotypes due to LIN-28 overexpression. This finding led us to identify 3′ UTR sequences that positively influence LIN-28 expression and balance repression by the *lin-4-CS* and *let-7-CS*. The *lin-28* 3′ UTR differs in this respect from the 3′ UTRs of the heterochronic genes *lin-14* and *hbl-1*, where deletion of most of their respective 3′ UTRs results in pronounced gain-of-function phenotypes resembling *lin-4* or *let-7* loss of function (Wightman et al., 1993; Ilbay and Ambros, 2019).

Mature *let-7* microRNA accumulates to higher levels as *C. elegans* development progresses, resulting from pulsed transcription of the *let-7* locus at each larval stage, despite LIN-28-mediated inhibition of mature *let-7* microRNA accumulation (Perales et al., 2014; Nelson and Ambros, 2019; Nahar et al., 2024). The concentration of *let-7* at the L4 stage (when *let-7* levels determine the fates of seam and vulval cells) presumably results from the integration of *let-7* biogenesis and turnover dynamics throughout larval development. Despite the superficially wild-type phenotype of *lin-28(*Δ*3′ UTR)* animals, we found that the dynamics of LIN-28 protein levels are abnormal – low in L1 samples and elevated in L4 samples. The elevated levels of LIN-28::GFP in *lin-28::GFP(*Δ*3′ UTR)* mutants during the L4 stage suggest partial inhibition of *let-7* microRNA production in this mutant at later developmental stages. Conversely, the lower levels of LIN-28::GFP at the L1 stage of *lin-28::GFP(*Δ*3′ UTR)* suggest increased *let-7* microRNA production during early larval stages. This implies that the wild-type phenotype of *lin-28(*Δ*3′ UTR)* mutants may reflect hyper-accumulation of *let-7* microRNA during early larval stages, which persists into later stages and may compensate for reduced *let-7* production caused by late-stage LIN-28 misexpression.

Introducing each of the three positive elements of the *lin-28* 3′ UTR into the *lin-*28*(*Δ*3′ UTR)* resulted in gain-of-function phenotypes, demonstrating that each element can independently promote LIN-28 activity without the remainder of the 3′ UTR and that these segments do not counteract the negative regulation imposed by the *let-7-CS* and *lin-4-CS*. Similarly, introducing the *let-7-CS* and *lin-4-CS* in *lin-28(*Δ*3′ UTR)* animals suppressed the *lin-28* gain-of-function phenotypes observed in the absence of *lep-5* lncRNA, indicating that these microRNA binding sites can function independently of the remaining 3′ UTR and do not counteract the positive regulatory elements.

Early L1 *lin-28(*Δ*3′ UTR)* animals exhibit ∼25% of the LIN-28 mRNA levels found in wild-type, indicating that specific regions within the *lin-28* 3′ UTR contribute to LIN-28 mRNA stabilization. Introducing one of the LIN-28-promoting 3′ UTR segments significantly increased LIN-28 mRNA levels compared to the *lin-28(*Δ*3′ UTR)* samples, suggesting that this element facilitates LIN-28 mRNA stabilization. However, this region alone did not fully restore LIN-28 mRNA levels, indicating that other regions, likely the two additional positive elements identified, further enhance mRNA stability. The mechanisms by which these 3′ UTR regions stabilize LIN-28 mRNA remain unclear and require further investigation.

In *C. elegans*, the *lin-28* locus produces two LIN-28 isoforms that differ in their N-termini (Seggerson et al., 2002; Moss and Tang, 2003). Both the N-termini and the C-terminus are predicted to be intrinsically disordered (Varadi et al., 2022; Abramson et al., 2024; UniProt Consortium, 2025). Many RNA-binding proteins have intrinsically disordered domains (IDRs) that are crucial for function (Ottoz and Berchowitz, 2020). Our data show that GFP-tagging of the LIN-28 C-terminus subtly impairs LIN-28 function, suggesting that the GFP tag may compromise an essential function of the C-terminal IDR.

Maintaining optimal expression levels of LIN-28 throughout larval development is essential for robust development and fecundity in *C. elegans*. In this study, we demonstrate that *C. elegans* have evolved multiple regulatory mechanisms – 3′ UTR-dependent regulation of LIN-28 production through the convergence of *lin-4* and *let-7* microRNAs and putative positive factors, as well as 3′ UTR-independent regulation of LIN-28 turnover by the LEP-2*/lep-5* lncRNA system – to ensure that LIN-28 is highly expressed during early larval stages and at appropriately lower levels during later larval stages, irrespective of environmental and physiological conditions. We hypothesize that this layered regulation of LIN-28 helps to ensure physiologically robust LIN-28 developmental dynamics. In support of this, *lin-28(*Δ*3′ UTR)* animals, which exhibit a wild-type phenotype under standard conditions, display retarded developmental phenotypes when exposed to *P. aeruginosa*. Likewise, the retarded phenotypes of *lin-28(*Δ*CSs)* are exacerbated by development on pathogenic bacterial food or at different temperatures. Further studies are required to elucidate how these modes of regulating LIN-28 may be coupled to physiological signals to robustly link LIN-28 developmental dynamics to developmental progression in wild-type animals.

## MATERIALS AND METHODS

### Nematode methods

*C. elegans* were cultured on nematode growth medium (NGM) (Brenner, 1974) and fed with *E. coli* HB101 unless otherwise noted. Synchronized populations of developmentally staged worms were obtained using standard methods (Stiernagle, 2006). A list of strains used in this study is in Table S3. Strains used in this study may be obtained from the *Caenorhabditis* Genetics Center (CGC), which is funded by the National Institutes of Health Office of Research Infrastructure Programs (P40 OD010440), or upon request from the corresponding author.

### Phenotypic analyses and scoring

For heterochronic phenotype analyses, one to four late L4 larvae were picked from healthy uncrowded cultures and placed onto individual NGM plates that were seeded with HB101. Animals were serially transferred daily for 3 days. Progeny on the plates were left to develop and scored for heterochronic phenotypes as young adults. Unless noted otherwise, all heterochronic phenotype analyses were performed at 15°C. Fluorescence microscopy was used to score COL-19::GFP. We note that adult phenotypic and COL-19::GFP scoring were not anonymized.

For adult (vulva function) phenotypic scoring (Fig. 2A), a score of 1 indicates adults that develop like wild-type, can actively lay eggs, and survive until a full brood is produced. A score of 2 indicates adults that are egg-laying defective and die due to a buildup of progeny within the mother. A score of 3 indicates an adult that dies due to bursting through a defective vulva after starting to produce progeny. A score of 4 indicates an adult that dies due to vulval bursting before producing progeny.

For COL-19::GFP scoring (Fig. 2B), a score of 1 indicates a wild-type expression pattern in the hyp-7 syncytium, seam cells, and vulva cells. A score of 2 indicates partial expression in the hyp-7 syncytium while maintaining wild-type-like expression in seam and vulva cells. A score of 3 indicates little to no expression in hyp-7 with wild-type-like expression in seam and vulva cells. A score of 4 indicates little to no expression in hyp-7, partial expression in seam cells, and wild-type-like expression in vulva cells. A score of 5 indicates expression only in vulva cells. A score of 6 indicates no apparent expression in any cell. Sample size (*n*) represents the number of animals assessed. These scoring descriptions comprehensively encompass the phenotypes of all animals observed in this study. We did not observe animals with phenotypes not matching these scoring descriptions (e.g. absent vulva expression with hyp-7/seam cell expression).

### Development on *Pseudomonas aeruginosa*

Development of *C. elegans* on *P. aeruginosa* was performed as previously described (Mirza et al., 2023).

### Microscopy and image quantification

All differential interference contrast (DIC) and fluorescent images were obtained using a W1-Yokogawa spinning disk Nikon confocal microscope equipped with a Prime BSI Express A22E726013 camera, a PLAN APO λD 60× OIL OFN25 DIC N2 objective, and the Nikon Elements software. Before imaging, worms were anesthetized with 0.2 mM levamisole in M9 buffer and mounted on 2% agarose pads. All LIN-28::GFP and pScm::GFP images were taken using the same microscopy settings. Pixel intensity of unmodified images was quantified using Fiji software. Adobe Photoshop was used to adjust the brightness and contrast of representative images for all figures to enhance the visualization of the DIC and fluorescent signals. Identical brightness and contrast adjustments were used for LIN-28::GFP fluorescent images that are displayed for comparison.

### *Caenorhabditis* genomes

All genomes used in this study were provided by the *Caenorhabditis* Genomes Project (doi:10.5281/zenodo.1263373).

### Phylogeny

*Caenorhabditis* phylogeny was adapted from Sun et al. (2022) and Picao-Osorio et al. (2025).

### Identification of *lin-28* homologs

Identification of *lin-28* homologs was performed using BLAST. All parameters were left at default settings (Camacho et al., 2009).

### 3′ UTR alignment and motif analysis

Alignment of probable *lin-28* 3′ UTRs (Table S1) was performed using MAFFT v7.505 with the L-INS-i algorithm (--localpair --maxiterate 1000) (Kuraku et al., 2013). Motif discovery was performed using MEME v5.5.9. Probable *lin-28* 3′ UTRs (Table S1) were analyzed in DNA mode with the classic expectation-maximization objective function. Motifs were identified using the zero-or-one occurrence per sequence (ZOOPS) model. Up to 20 motifs were searched, with motif widths restricted to 4-10 nucleotides. Background nucleotide frequencies were estimated using a zeroth-order Markov model. All other parameters were left at default settings (Bailey and Elkan, 1994).

### RNA extraction

Populations of animals were grown at 20°C, collected in M9 buffer, flash-frozen in liquid nitrogen, and total RNA was extracted by adding ∼20 μl of 100 μm zirconium beads (OPS Diagnostics) and homogenized in a Next Advance Bullet Blender Blue at setting 8 for 2 min at 4°C with Qiazol reagent (Qiagen). The remainder of the RNA extraction protocol is as described in McJunkin and Ambros (2017).

### Protein extraction

Populations of animals were grown at 20°C, collected in M9 buffer, and flash-frozen in liquid nitrogen. Protein was extracted by adding an equal volume of 2× Laemmli buffer without dye [20% glycerol, 4% SDS, 0.125 M Tris (pH 6.8), and 0.1 M DTT] and ∼20 μl of 100 μm zirconium beads (OPS Diagnostics), homogenized in a Next Advance Bullet Blender Blue at setting 8 for 2 min at 4°C, heated for 5 min at 99°C, centrifuged at 16.1 ***g*** for 5 min at room temperature, and the supernatant was transferred to a fresh tube.

### Western blotting

Western blotting was performed as described previously (Dutta and Baehrecke, 2008). Primary antibodies used were rabbit anti-LIN-28 (1:10,000; Seggerson et al., 2002) and mouse anti-alpha-tubulin (1:10,000; Sigma-Aldrich, T6074). Western blots were imaged using Amersham Imager 600.

It is important to note that our western blot analyses using the anti-LIN-28 antibody displayed bands that migrated inconsistently with the expected molecular weight of LIN-28 isoforms; none of these bands exhibited a shift in size in samples containing LIN-28::GFP, indicating that they are cross-reacting peptides unrelated to LIN-28. To further validate this observation, we employed CRISPR/Cas9 to delete the entire coding sequence of *lin-28*, resulting in the loss of the two bands presumed to correspond to LIN-28A and LIN-28B, while the suspected non-specific bands remained [*lin-28(ma637)*; Fig. S1C].

### Quantitative PCR

For all mRNA quantification, cDNA was synthesized using SuperScript IV (Invitrogen) following the manufacturer's instructions. For all small RNA quantification, cDNA was synthesized using miRNA 1st Strand cDNA Synthesis Kit (by stem-loop) (Vazyme) following the manufacturer's instructions. qPCR reactions were performed using 2× UltraSYBR Mixture (Low ROX) (CoWin Biosciences) following the manufacturer's instructions, using a QuantStudio 12K Flex (Applied Biosystems). For all LIN-28 mRNA experiments, ΔCTs were calculated by normalizing samples to ACT-1/2 mRNA. For all *let-7* microRNA experiments, ΔCTs were calculated by normalizing samples to U18 snoRNA. Sample size (*n*) represents the number of biological replicates used. For each biological replicate, the average of three technical replicates was used. All oligos used for cDNA synthesis and qPCR in this study are described in Table S4.

### Transgenic constructs

The extrachromosomal array maEx268, which was used to rescue *lep-5* in the strains VT4303 and VT4307, was generated by PCR cloning the *lep-5* locus. The resulting PCR product was column purified and injected along with the *rol-6(su1006)*-containing plasmid pRF-4 (Mello et al., 1991) into *lep-5(ma613)* mutants. The extrachromosomal array maEx269, which was used to rescue *lep-5* in the strains VT4400, VT4411, VT4412 and VT4413, was generated by PCR cloning the *lep-5* locus, and by PCR cloning *rol-6(su1006)* from the plasmid pRF-4 and *Pmyo-2::mCherry* from the Addgene plasmid pCFJ90. The resulting PCR products were column purified and injected into *lep-5(ma613)* mutants. All oligos used to generate transgenic constructs in this study are described in Table S4.

### Targeted genome editing by CRISPR/Cas9

Mutants were generated using CRISPR/Cas9 methods adapted from Nelson and Ambros, (2019, 2021). All *lin-28* 3′ UTR alleles generated to identify positive regulatory elements used a ‘jump-board’ in which most of the 3′ UTR was replaced with an artificial CRISPR landing site used for inserting truncated versions of the *lin-28* 3′ UTR (Fig. S2A) (Duan et al., 2020). See Table S5 for a detailed list of alleles and Table S4 for descriptions of the oligos used in allele generation in this study. All alleles listed with two single-stranded oligodeoxynucleotides (ssODNs) as HR templates used two ssODNs with overlapping complementarity and singled stranded 5′ overhangs in the generation of the allele.

### Use of artificial intelligence tools

EditGPT was used to aid in the grammatical editing of this manuscript. After using this service, the authors reviewed and edited the content as needed and take full responsibility for the content of the publication.

## Supplementary Material



10.1242/develop.205391_sup1 Supplementary information

Table S1. lin-28 3' UTRs in *Caenorhabditis* species have conserved *let-7* and *lin-4* CSs. List of all the *Caenorhabditis* species used in this study along with their predicted lin-28 isoforms, predicted 3' UTRs, and predicted *let-7* and *lin-4* CSs.

Table S2. lin-28 3' UTRs in *Caenorhabditis* species have conserved sequence motifs. List of the top 20 conserved motifs found in the predicted lin-28 3' UTRs of *Caenorhabditis* species analyzed in this study.

Table S3. *C. elegans* strains used in this study. List of all the *C. elegans* strains, their genotypes, related figure(s), and descriptions used in this study.

Table S4. Oligos used in this study. List and description of oligos used for the generation of CRISPR mutants, qPCR analyses, and transgenics in this study.

Table S5. Endogenous C. elegans alleles generated for this study. List and description of all the endogenous *C. elegans* CRISPR alleles including genomic sequences and methods used for their generation.
