# Supplementary material for: Multi-dimensional regulation of LIN-28 temporal expression dynamics in the C. elegans heterochronic gene cascade
Source: Development. 2026 May 28;153(10):dev205391. doi: 10.1242/dev.205391 (PMC13286352; doi:10.1242/dev.205391)
Supplement: Supplementary information [file develop-153-205391-s1.pdf]

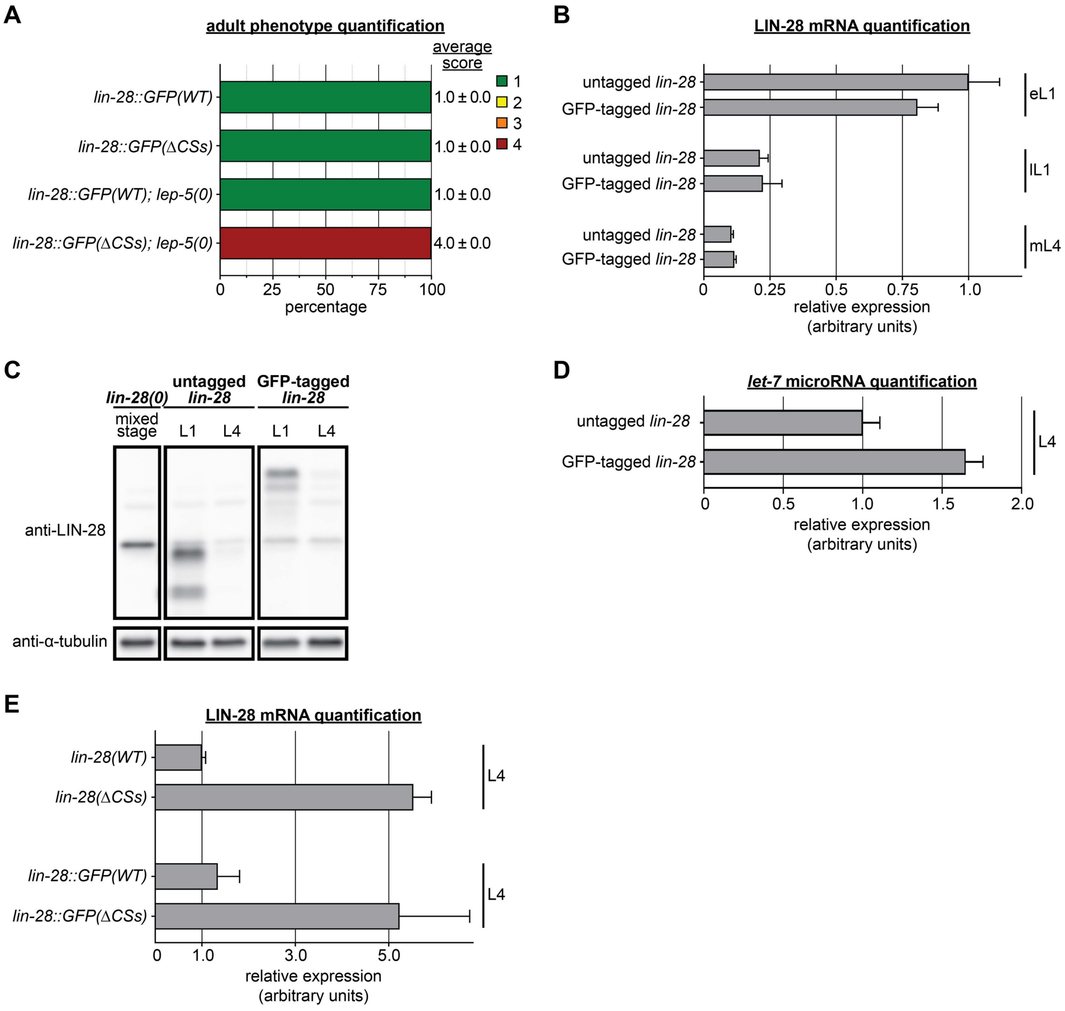

Fig. S1. GFP tagging impairs the function of LIN-28.

(A) Quantification of adult phenotypes of *lin-28::GFP* animals with either a wild-type 3' UTR or ΔCSs in the presence and absence of *lep-5*. N's (from top to bottom) = 52, 99, 74, and 51. Data are presented as mean score ± standard deviation.

(B) RT-qPCR analysis of LIN-28 mRNA in whole animal extracts from early L1, late L1, and mid L4 stages of wild-type untagged and GFP-tagged *lin-28* strains. N = 4. Data are presented as mean ± standard deviation.

(C) Whole animal lysates from *lin-28* null animals (mixed stage sample), untagged LIN-28 (L1 and L4 samples), and GFP-tagged LIN-28 (L1 and L4 samples) were analyzed using western blotting with an anti-LIN-28 antibody.

(D) RT-qPCR analysis of *let-7* microRNA levels in mid L4 stage whole animal extracts of wild-type untagged and GFP-tagged *lin-28* strains. N = 5. Data are presented as mean ± standard deviation.

(E) RT-qPCR analysis of LIN-28 mRNA in whole animal extracts from L4 wild-type untagged and GFP-tagged *lin-28* strains. N = 3. Data are presented as mean ± standard deviation.

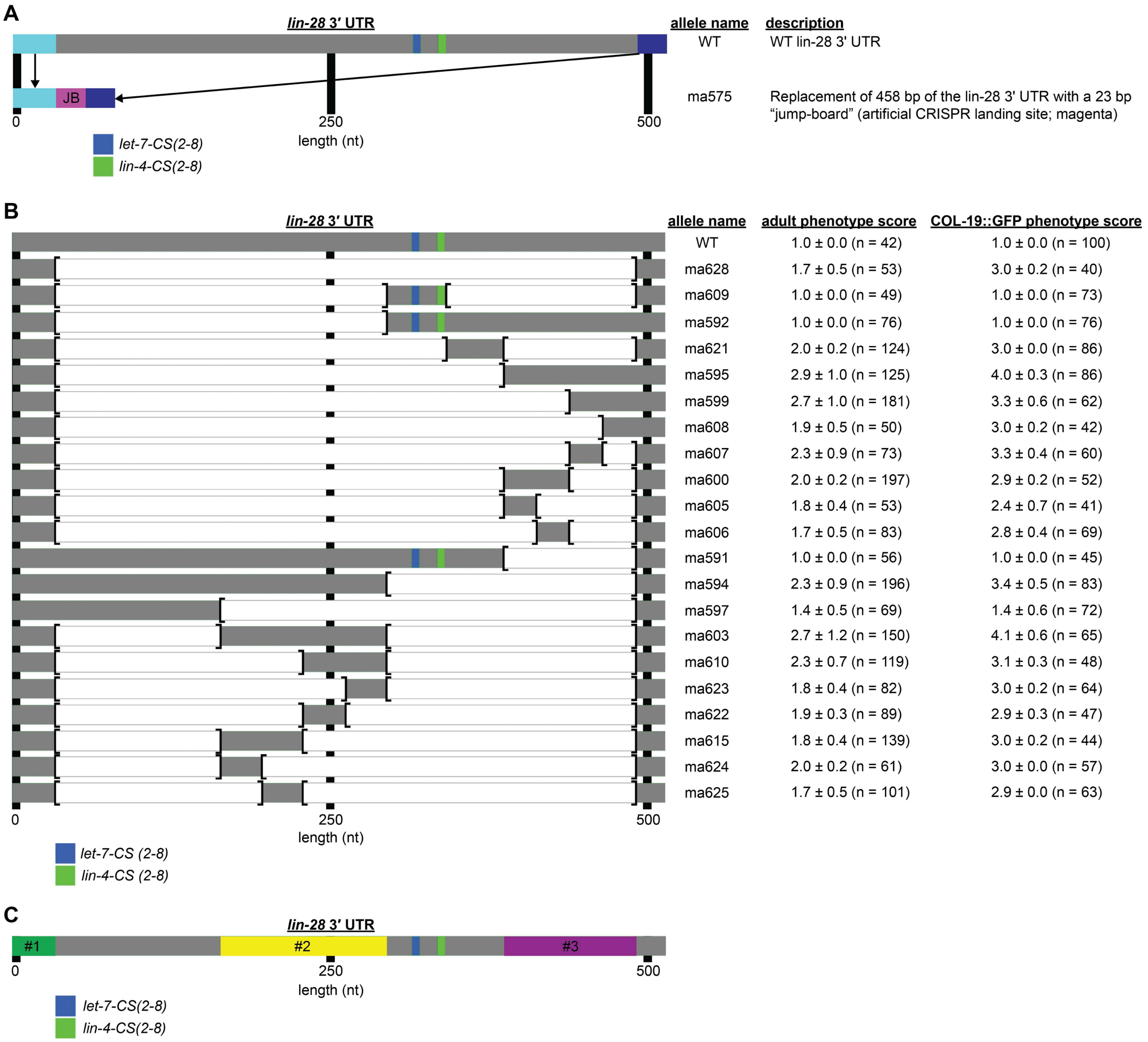

Fig. S2. 3' UTR truncations of *lin-28* generated to identify positive regulatory elements.

(A) Depiction of the wild-type *C. elegans lin-28* 3' UTR and *lin-28(ma575)*, which replaces 458 bp of the endogenous *lin-28* 3' UTR with the “jump-board” (JB) sequence (artificial CRISPR landing site; magenta) (Duan et al. 2020).

(B) Depiction of the endogenous *lin-28* 3' UTR truncation strains generated to isolate regions that contain positive regulatory elements and quantifications of adult and COL-19::GFP phenotype scores. Data are presented as mean score ± standard deviation.

(C) Depiction of the wild-type *C. elegans lin-28* 3' UTR with positive regulatory element regions show in green (positive element #1), yellow (positive element #2), and purple (positive element #3).

Development • Supplementary information

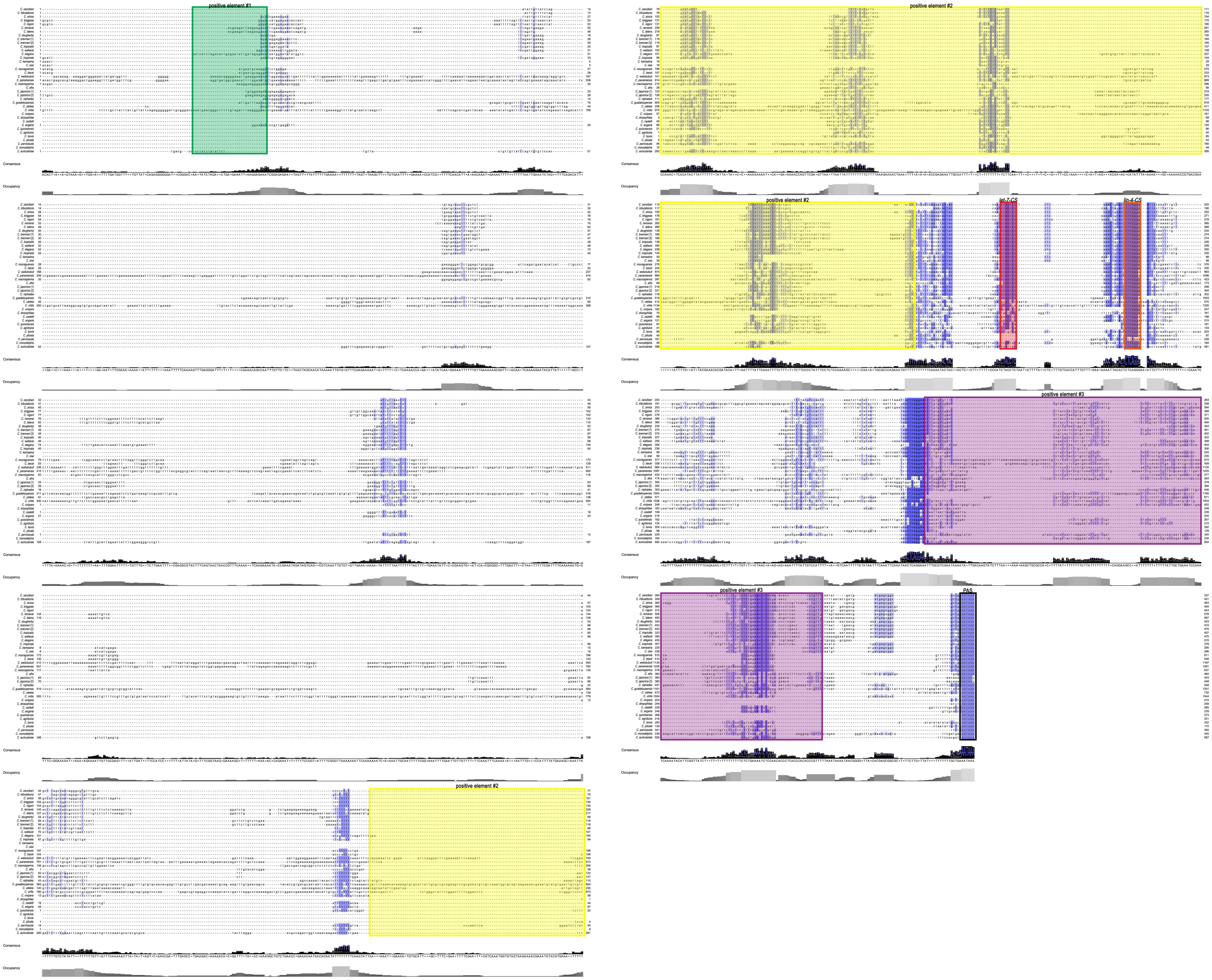

Fig. S3. Regions of conserved sequence can be found throughout the *lin-28* 3' UTRs in *Caenorhabditis* species.

Alignment of all predicted *lin-28* 3' UTRs in *Caenorhabditis* species analyzed in this study show conserved regions can be found in positive element #2 (*C. elegans* region highlighted in yellow) and positive element #3 (*C. elegans* region highlighted in purple), but not in positive element #1 (*C. elegans* region highlighted in green). The *C. elegans let-7-CS* and *lin-4-CS* are highlighted in red and orange, respectively. The polyadenylation signal (PAS) is outlined in black.

Development • Supplementary information

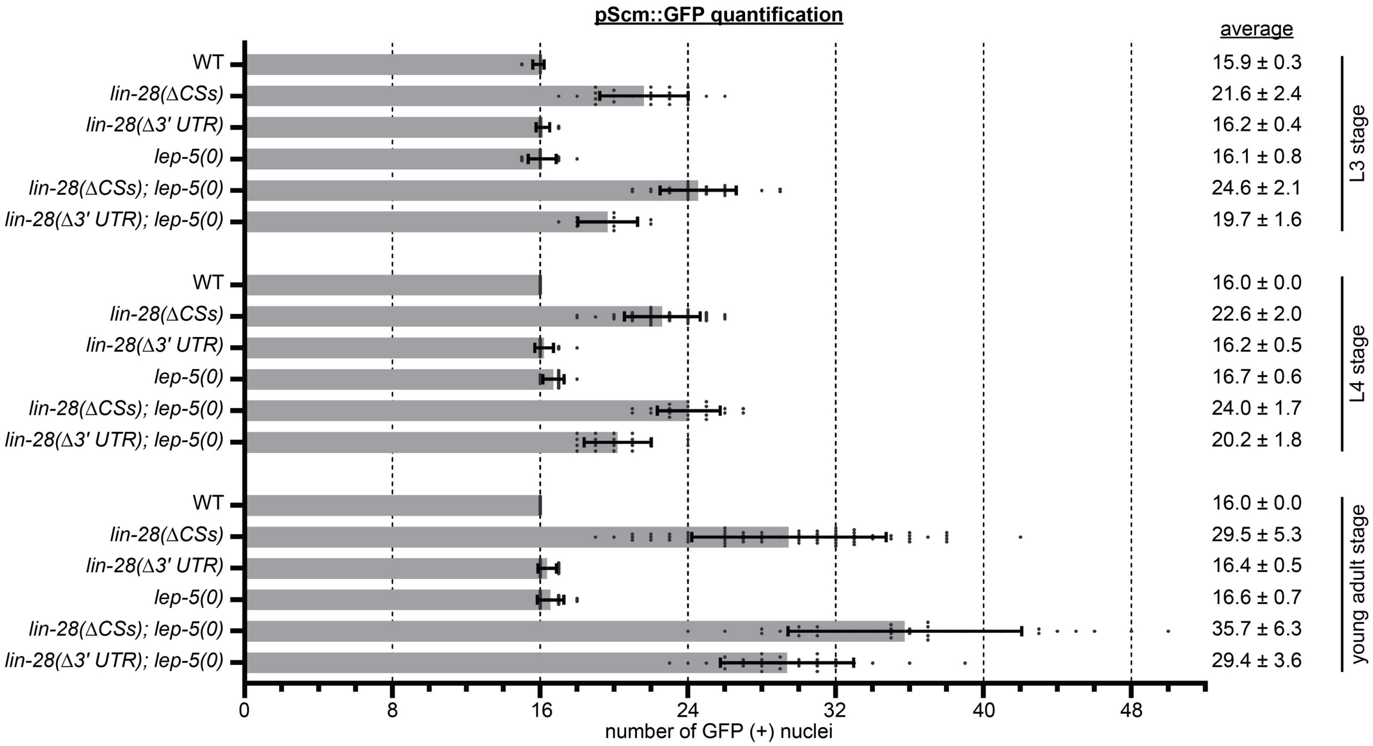

Fig. S4. *C. elegans* strains with *lin-28* gain-of-function phenotypes have more seam cells than strains with no apparent gain-of-function phenotypes.

Quantification of pScm::GFP-positive nuclei in L3-stage, L4-stage, and young adult wild-type, *lin-28(ΔCSs)*, and *lin-28(Δ3' UTR)* animals with and without *lep-5*. N's (from top to bottom) = 21, 24, 20, 18, 37, 12, 26, 42, 27, 18, 22, 20, 23, 68, 26, 28, 35, and 27.

Development • Supplementary information

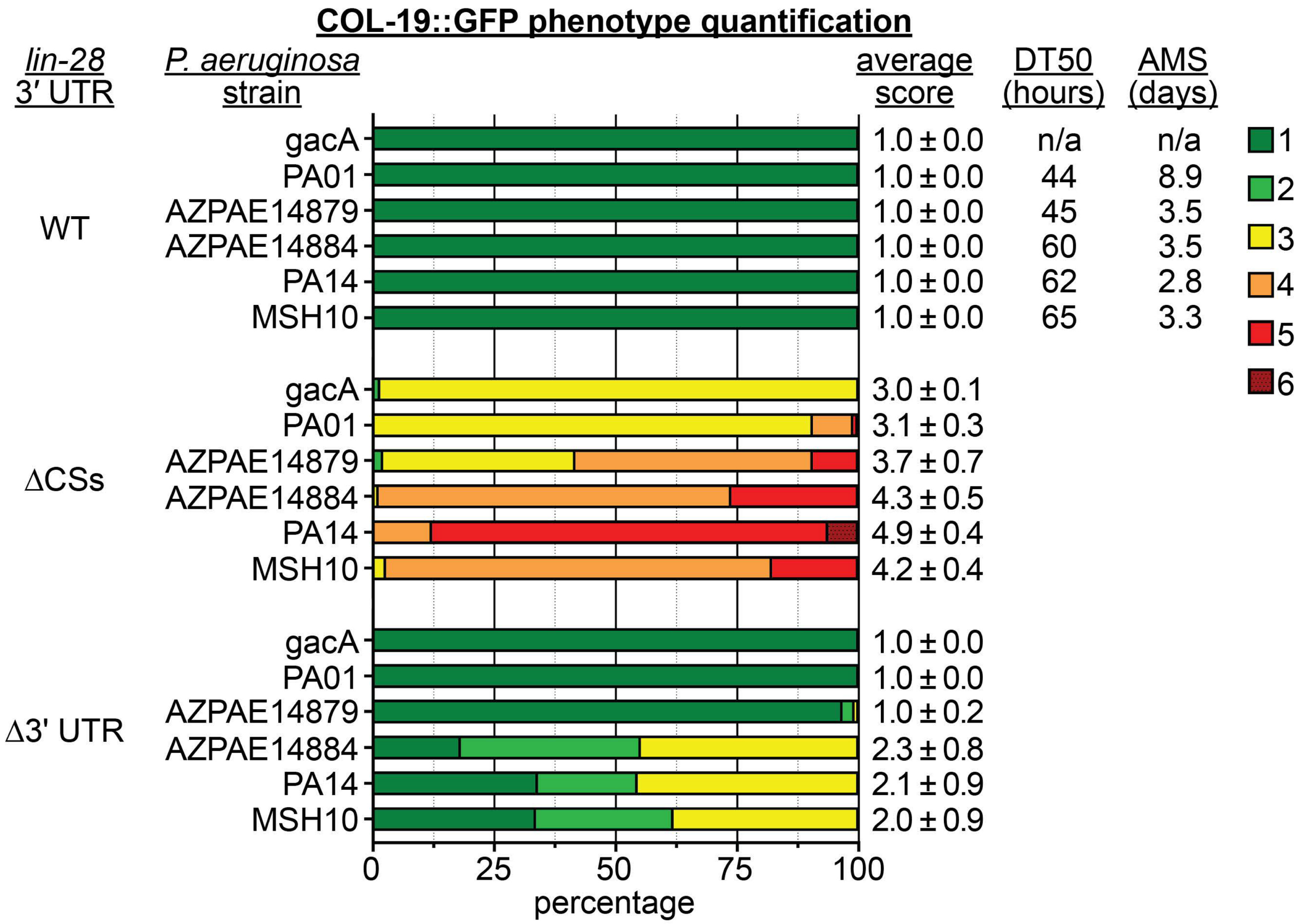

Fig. S5. Both virulence and developmental slowing exacerbate *lin-28* gain-of-function phenotypes.

Quantification of COL-19::GFP phenotypes of animals exposed to *P. aeruginosa* strains that vary in their virulence (AMS) and effect on developmental rate (DT50). PA14 and gacA data are the same as Fig. 7A. N's (from top to bottom) = 98, 107, 123, 72, 44, 55, 66, 96, 235, 84, 49, 73, 107, 165, 121, 105, 44, and 113. DT50 and AMS data are from (Mirza et al. 2023).

Development • Supplementary information

Table S1. *lin-28* 3' UTRs in *Caenorhabditis* species have conserved *let-7* and *lin-4* CSs. List of all the *Caenorhabditis* species used in this study along with their predicted *lin-28* isoforms, predicted 3' UTRs, and predicted *let-7* and *lin-4* CSs.

Available for download at
https://journals.biologists.com/dev/article-lookup/doi/10.1242/dev.205391#supplementary-data

Table S2. *lin-28* 3' UTRs in *Caenorhabditis* species have conserved sequence motifs. List of the top 20 conserved motifs found in the predicted *lin-28* 3' UTRs of *Caenorhabditis* species analyzed in this study.

Available for download at
https://journals.biologists.com/dev/article-lookup/doi/10.1242/dev.205391#supplementary-data

Table S3. *C. elegans* strains used in this study.
List of all the *C. elegans* strains, their genotypes, related figure(s), and descriptions used in this study.

Available for download at
https://journals.biologists.com/dev/article-lookup/doi/10.1242/dev.205391#supplementary-data

Table S4. Oligos used in this study.
List and description of oligos used for the generation of CRISPR mutants, qPCR analyses, and transgenics in this study.

Available for download at
https://journals.biologists.com/dev/article-lookup/doi/10.1242/dev.205391#supplementary-data

Table S5. Endogenous *C. elegans* alleles generated for this study.
List and description of all the endogenous *C. elegans* CRISPR alleles including genomic sequences and methods used for their generation.

Available for download at
https://journals.biologists.com/dev/article-lookup/doi/10.1242/dev.205391#supplementary-data

Development • Supplementary information
