# Supplementary material for: Multi-dimensional regulation of LIN-28 temporal expression dynamics in the C. elegans heterochronic gene cascade
Source: Development. 2026 May 28;153(10):dev205391. doi: 10.1242/dev.205391 (PMC13286352; doi:10.1242/dev.205391)
Supplement: Supplementary file 1 [file develop-153-205391_review_history.pdf]

Multi-dimensional regulation of LIN-28 temporal expression dynamics in the *C. elegans* heterochronic gene cascade

Charles Nelson and Victor Ambros
DOI: 10.1242/dev.205391

Editor: Swathi Arur

Review timeline

Original submission:	1 December 2025
Editorial decision:	5 January 2026
First revision received:	19 March 2026
Accepted:	22 April 2026

Original submission

First decision letter

MS ID#: dev.205391

MS TITLE: Multi-dimensional regulation of LIN-28 temporal expression dynamics in the C. elegans heterochronic gene cascade

AUTHORS: Victor Ambros; Charles Nelson

Dear Dr Nelson,

I have now received all the referees reports on the above manuscript, and have reached a decision. The referees' comments are appended below.

The overall evaluation is positive and we would like to publish a revised manuscript in Development. However, the referees make recommendations for clarity and increased. Please attend to all of the reviewers' comments in your revised manuscript and detail them in your point-by-point response. If you do not agree with any of their criticisms or suggestions explain clearly why this is so.

Reviewer 1

Review of "Multi-dimensional regulation of LIN-28 temporal expression dynamics in the C. elegans heterochronic gene cascade"

Summary

This manuscript substantially refines our understanding of LIN-28 regulation within the heterochronic pathway by uncovering multiple, parallel mechanisms that converge on the lin-28 3'UTR and LIN-28 protein to shape its developmental expression dynamics. Using precise genome engineering at the endogenous lin-28 locus, the authors identify several key regulatory principles.

First, binding sites for lin-4 and let-7-family microRNAs are shown to be functionally redundant yet jointly required for proper LIN-28 repression. This result clarifies long-standing ambiguities arising

© 2026. Published by The Company of Biologists under the terms of the Creative Commons Attribution License (https://creativecommons.org/licenses/by/4.0/).

from earlier studies (e.g., Moss et al., 1997) that relied on high-copy extrachromosomal arrays and establishes a convergent role for these microRNAs in lin-28 control.

Second, lin-4 and let-7-family microRNAs act in parallel with an additional non-coding RNA, lep-5, that regulates LIN-28 protein levels independently of the lin-28 3′UTR, revealing a previously unappreciated layer of post-transcriptional control.

Third, and most unexpectedly, the authors demonstrate that the native lin-28 3 ′ UTR is dispensable for normal LIN-28 regulation under standard conditions. These experiments are convincing and imply that negative regulatory inputs are counterbalanced by positive cis-elements within the endogenous 3′UTR. The subsequent identification of these elements strengthens this conclusion.

Fourth, the manuscript shows that GFP tagging perturbs LIN-28 function and regulation, raising a broader concern for the field: many heterochronic genes with dynamic expression patterns have been characterized almost exclusively using fluorescent fusions, which may not faithfully report endogenous regulation.

Finally, the authors show that the lin-28 3′UTR contributes to robustness under non-standard laboratory conditions, underscoring that much of what is known about the heterochronic pathway derives from artificial environments and that additional regulatory layers likely remain unexplored.

Collectively, this is a solid and complete work. These findings make a substantive contribution to our understanding of temporal patterning and regulatory redundancy in developmental gene networks and would be suitable for Development as an article as is, with only minor edits.

Minor critique

The authors employ two scoring rubrics to quantify penetrance of lin-28(gf) phenotypes, primarily based on terminal developmental outcomes. While useful for comparing complex genotypes, this approach misses the opportunity to pinpoint when, during development, defects first arise. Earlier work using extrachromosomal LIN-28 reporters (e.g., Moss et al., 1997) suggested reiteration of L2 seam cell divisions. It would be informative to assess whether comparable lineage defects occur in selected endogenous mutants. A limited lineage analysis of one or two severe genotypes (e.g., lin-28(ΔCS) versus lin-28(ΔCS); lep-5(0)) would substantially clarify the cellular basis of the observed phenotypes without imposing an unreasonable experimental burden.

Typographical corrections
* Page 5: Missing space after the period in "(Fig. 1 and Table S1).All 3′ UTR regions…"
* Page 6: Missing space in "downregulateLIN-28."
* Figure 4: Panel a contains an incorrect color after the #3 enhancer element (yellow should be red).

<u>Reviewer 2</u>

SUMMARY OF THE ADVANCE MADE IN THIS PAPER AND ITS POTENTIAL SIGNIFICANCE TO THE FIELD

This paper investigates the regulation of the conserved RNA-binding protein (RBP) LIN-28 via its 3'UTR in C. elegans, also elucidating how misregulation as a consequence of altered LIN-28 3' UTR causes aberrant expression of the let-7 microRNA (miRNA) downstream.

The study uses three main methods: 1) CRISPR/Cas9 genome editing to systematically delete parts of the 3' UTR sequence 2) phenotypic scoring of classic C. elegans heterochronic phenotypes (egg-laying defects, vulval bursting) as we as qualitative scoring of the expression patterns of LIN-28 GFP 3) RT-qPCR to detect LIN-28 and let-7 levels in various backgrounds with altered 3'UTR sequences.

The main findings of this paper are: 1) Deleting largely conserved binding sites in the LIN-28 3'UTR for either let-7 or lin-4 separately has only minor phenotypic effects, whereas they deletion of both leads to retarded phenotypes (similar to let-7 mutant phenotypes), exacerbated by removing the long non-coding RNA lep-5.

© 2026. Published by The Company of Biologists under the terms of the Creative Commons Attribution License (https://creativecommons.org/licenses/by/4.0/).

2) These phenotypes are likely caused lack of LIN-28 downregulation, leading to reduced levels let-7 at the L4 stage.
3) Surprisingly, remove the entire 3'UTR does not phenotypic deletion of the miRNA binding sites alone, but rather reduces lin-28 mRNA levels early in development compared to WT and evaluates them compared to WT late in larval development. Overall, these animals are phenotypically WT, likely because LIN-28 protein is ultimately downregulated by lep-5 late in larval development. Through a series of 3'UTR truncations, the authors identify a specific region of the 3'UTR that positively regulates LIN-28 mRNA levels.
4) Finally, the authors show that the combination of positive and negative regulations of lin-28 enhances robustness of temporal stem cells patterning, with respect to different growth temperatures and growth on pathogenic bacteria.

This is an important paper in the field of developmental timing and adds considerable insight about the regulation of a conserved RBP by the miRNAs. It is meticulously executed and the conclusion are justified by the data. I only have minor comments to make and, after addressing those, I think the paper is highly suitable for Development.

SUGGESTIONS TO AUTHORS

Minor Comments:

Figure 1: Do the authors have any explanation for why there are no let-7 sites in those distantly related species? Those species do have let-7/mir-84 typically, right?

The color scheme in this figure is hard to read. Could you authors make the grey bars lighter to enhance visibility of the sites?

Classification of lin-28 phenotypes/Figure 2:
While the adult phenotype classification appears rather straightforward to apply objectively (e.g. no egg-laying, egg-laying), the col-19::GFP phenotypes are rather continuous. There positioning in the classes appears somewhat subjective. E.g., is vulval expression ever absent, when hyp and seam are still expressing?

The methods section suggests that scoring was NOT done blindly (i.e., genotypes not known to the person scoring phenotypes), which could lead to biases in the results). Can the authors repeat at least key parts (e.g., Fig. 2e,4c) blindly to confirm that this didn't happen?

Could the authors add a panel to figure S2, drawing the three regions that positively regulated lin-28 mRNA. It is not completely straightforward to infer this from the series of truncations they did and their phenotypes, and it would help cut to the chase here.

Could the authors determine whether the 3' UTR sequences required for positive regulation of LIN-28 are conserved among other nematode species?

For the figure 7, can the authors determine whether the animals on PA14 grow slower than those on gacA? In other words, is it possible that the PA14 phenotype and the 15°C phenotypes are both resulting from the fact that the animals are unable to slow down lin-28 gene expression dynamics appropriately?

First revision

Author response to reviewers' comments

Response to reviews - Nelson and Ambros

Reviewer 1 Summary:

© 2026. Published by The Company of Biologists under the terms of the Creative Commons Attribution License (https://creativecommons.org/licenses/by/4.0/).

This manuscript substantially refines our understanding of LIN-28 regulation within the heterochronic pathway by uncovering multiple, parallel mechanisms that converge on the lin-28 3′UTR and LIN-28 protein to shape its developmental expression dynamics. Using precise genome engineering at the endogenous lin-28 locus, the authors identify several key regulatory principles.

First, binding sites for lin-4 and let-7-family microRNAs are shown to be functionally redundant yet jointly required for proper LIN-28 repression. This result clarifies long-standing ambiguities arising from earlier studies (e.g., Moss et al., 1997) that relied on high-copy extrachromosomal arrays and establishes a convergent role for these microRNAs in lin-28 control.

Second, lin-4 and let-7-family microRNAs act in parallel with an additional non-coding RNA, lep-5, that regulates LIN-28 protein levels independently of the lin-28 3′UTR, revealing a previously unappreciated layer of post-transcriptional control.

Third, and most unexpectedly, the authors demonstrate that the native lin-28 3 ′ UTR is dispensable for normal LIN-28 regulation under standard conditions. These experiments are convincing and imply that negative regulatory inputs are counterbalanced by positive cis-elements within the endogenous 3′UTR. The subsequent identification of these elements strengthens this conclusion.

Fourth, the manuscript shows that GFP tagging perturbs LIN-28 function and regulation, raising a broader concern for the field: many heterochronic genes with dynamic expression patterns have been characterized almost exclusively using fluorescent fusions, which may not faithfully report endogenous regulation.

Finally, the authors show that the lin-28 3′UTR contributes to robustness under non-standard laboratory conditions, underscoring that much of what is known about the heterochronic pathway derives from artificial environments and that additional regulatory layers likely remain unexplored.

Collectively, this is a solid and complete work. These findings make a substantive contribution to our understanding of temporal patterning and regulatory redundancy in developmental gene networks and would be suitable for Development as an article as is, with only minor edits.

Reviewer 1 Comments:

1. The authors employ two scoring rubrics to quantify penetrance of lin-28(gf) phenotypes, primarily based on terminal developmental outcomes. While useful for comparing complex genotypes, this approach misses the opportunity to pinpoint when, during development, defects first arise. Earlier work using extrachromosomal LIN-28 reporters (e.g., Moss et al., 1997) suggested reiteration of L2 seam cell divisions. It would be informative to assess whether comparable lineage defects occur in selected endogenous mutants. A limited lineage analysis of one or two severe genotypes (e.g., lin- 28(ΔCS) versus lin-28(ΔCS); lep-5(0)) would substantially clarify the cellular basis of the observed phenotypes without imposing an unreasonable experimental burden.

    Response:

    This is a good suggestion. We crossed in the seam cell GFP reporter (pScm::GFP) into *lin-28(ΔCSs)*, *lin-28(Δ3' UTR)*, *lep-5(0)*, and their respective double mutants and counted **seam cell numbers in L3, L4, and young adult animals. These results were informative and suggest reiteration of L2 seam cell divisions as reviewer 1 hypothesized. These data have been added to the results section, and an additional supplemental figure (Fig. S3) has been generated to show the data.**

2. Typographical corrections
    a. Page 5: Missing space after the period in "(Fig. 1 and Table S1).All 3′ UTR regions…"

© 2026. Published by The Company of Biologists under the terms of the Creative Commons Attribution License (https://creativecommons.org/licenses/by/4.0/).

b. Page 6: Missing space in "downregulateLIN-28."
c. Figure 4: Panel a contains an incorrect color after the #3 enhancer element (yellow should be red).

Response:

Thank you for these observations. We have corrected them.

Reviewer 2 Summary:

This paper investigates the regulation of the conserved RNA-binding protein (RBP) LIN-28 via its 3'UTR in C. elegans, also elucidating how misregulation as a consequence of altered LIN-28 3' UTR causes aberrant expression of the let-7 microRNA (miRNA) downstream.

The study uses three main methods: 1) CRISPR/Cas9 genome editing to systematically delete parts of the 3' UTR sequence 2) phenotypic scoring of classic C. elegans heterochronic phenotypes (egg-laying defects, vulval bursting) as we as qualitative scoring of the expression patterns of LIN-28 GFP 3) RT-qPCR to detect LIN-28 and let-7 levels in various backgrounds with altered 3'UTR sequences.

The main findings of this paper are:

1. Deleting largely conserved binding sites in the LIN-28 3'UTR for either let-7 or lin-4 separately has only minor phenotypic effects, whereas they deletion of both leads to retarded phenotypes (similar to let-7 mutant phenotypes), exacerbated by removing the long non- coding RNA lep-5.

2. These phenotypes are likely caused lack of LIN-28 downregulation, leading to reduced levels let-7 at the L4 stage.

3. Surprisingly, remove the entire 3'UTR does not phenotypic deletion of the miRNA binding sites alone, but rather reduces lin-28 mRNA levels early in development compared to WT and evaluates them compared to WT late in larval development. Overall, these animals are phenotypically WT, likely because LIN-28 protein is ultimately downregulated by lep-5 late in larval development.

Through a series of 3'UTR truncations, the authors identify a specific region of the 3'UTR that positively regulates LIN-28 mRNA levels.

4. Finally, the authors show that the combination of positive and negative regulations of lin-28 enhances robustness of temporal stem cells patterning, with respect to different growth temperatures and growth on pathogenic bacteria.

This is an important paper in the field of developmental timing and adds considerable insight about the regulation of a conserved RBP by the miRNAs. It is meticulously executed and the conclusion are justified by the data. I only have minor comments to make and, after addressing those, I think the paper is highly suitable for Development.

Reviewer 2 Comments:

1. Figure 1: Do the authors have any explanation for why there are no let-7 sites in those distantly related species? Those species do have let-7/mir-84 typically, right?

Response:

We have expanded the discussion section to include more on this topic. However, due to word constraints, we did not significantly elaborate further. While this is a

© 2026. Published by The Company of Biologists under the terms of the Creative Commons Attribution License (https://creativecommons.org/licenses/by/4.0/).

fascinating topic, we believe that a more extensive discussion is beyond the scope of this study.

2. The color scheme in this figure is hard to read. Could you authors make the grey bars lighter to enhance visibility of the sites?

Response:

We have lightened the gray bars in Figure 1 to reflect this suggestion.

3. Classification of lin-28 phenotypes/Figure 2:

While the adult phenotype classification appears rather straightforward to apply objectively (e.g. no egg-laying, egg-laying), the col-19::GFP phenotypes are rather continuous. There positioning in the classes appears somewhat subjective. E.g., is vulval expression ever absent, when hyp and seam are still expressing?

Response:

We appreciate this perspective. By nature, the col-19::GFP phenotypes are more fluid, but we believe this scoring method accurately reflects our observations and improves on previously published descriptions of this phenomenon. While there may be phenotypes that do not match our descriptions (e.g. absent vulva expression with hyp-7/seam cell expression), we did not observe these. If we had observed anything different, we would have noted it in our results. We have added a note in the materials and methods to emphasize this.

4. The methods section suggests that scoring was NOT done blindly (i.e., genotypes not known to the person scoring phenotypes), which could lead to biases in the results). Can the authors repeat at least key parts (e.g., Fig. 2e,4c) blindly to confirm that this didn't happen?

Response:

Our scoring was not done blindly, but we believe this did not impact our study. Our scoring method is consistent with previous publications, and the phenotypic comparisons made (e.g., in Fig. 2e and 4c) between genotypes were significantly different enough that blind comparisons were unnecessary. Therefore, we did not repeat these experiments blindly.

5. Could the authors add a panel to figure S2, drawing the three regions that positively regulated lin-28 mRNA. It is not completely straightforward to infer this from the series of truncations they did and their phenotypes, and it would help cut to the chase here.

Response:

We have added this suggested panel to Fig. S2.

6. Could the authors determine whether the 3' UTR sequences required for positive regulation of LIN-28 are conserved among other nematode species?

Response:

This is a good suggestion. We performed alignment and motif analyses of the *lin-28* 3' UTR homologs from Fig. 1 and added these results in a new supplemental figure and supplemental table (Fig. S3 and Table S3). A paragraph describing these data has been added to the results section. Please note that all subsequent supplemental table numbers have been adjusted accordingly (previous S3 is now S4, etc.).

7. For the figure 7, can the authors determine whether the animals on PA14 grow slower

© 2026. Published by The Company of Biologists under the terms of the Creative Commons Attribution License (https://creativecommons.org/licenses/by/4.0/).

than those on gacA? In other words, is it possible that the PA14 phenotype and the 15˚C phenotypes are both resulting from the fact that the animals are unable to slow down lin-28 gene expression dynamics appropriately?

Response:

This is a good question. To address this, we exposed wild-type, *lin-28(ΔCSs)*, and *lin-28(Δ3' UTR)* mutants to four additional *P. aeruginosa* strains that differ in developmental rate slowing and virulence. Our results indicate that both factors contribute to the exacerbated phenotypes. Paragraphs addressing this have been added to the results section, along with a new supplemental figure (Fig. S5) to show the data.

Second decision letter

MS ID#: dev.205391R1

MS TITLE: Multi-dimensional regulation of LIN-28 temporal expression dynamics in the C. elegans heterochronic gene cascade

AUTHORS: Victor Ambros; Charles Nelson
ARTICLE TYPE: Research Article

Dear Dr Nelson,

I am happy to tell you that your manuscript has been accepted for publication in Development, pending our standard publication integrity checks.

Reviewer 2

I am happy with the changes made to the manuscript and I recommend publishing without any further modifications. Congratulations to all the authors for this wonderful work!

© 2026. Published by The Company of Biologists under the terms of the Creative Commons Attribution License (https://creativecommons.org/licenses/by/4.0/).
